# Anisotropic Charge Diffusion in Polar‐Layered Oxides for Ultralong Charge Retention

**DOI:** 10.1002/advs.202514554

**Published:** 2025-10-08

**Authors:** Sungjun Choi, Yujin Choi, Jeongdae Seo, Sanghyeok Ryou, Minwoo Jang, Haeyun Song, Sanghoon Yeom, Hyungwoo Lee

**Affiliations:** ^1^ Department of Energy Systems Research Ajou University Suwon 16499 Republic of Korea; ^2^ Ajou Energy Science Research Center Ajou University Suwon 16499 Republic of Korea; ^3^ Department of Physics Ajou University Suwon 16499 Republic of Korea

**Keywords:** anisotropic charge diffusion, charge retention, defect‐mediated hopping, LaAlO_3_, oxygen vacancies

## Abstract

Persistent surface charge retention in dielectric oxides is critical for a wide range of electronic and energy applications, including charge‐trapping memory devices, triboelectric generators, and supercapacitors. Since charge retention is intrinsically governed by charge diffusion, understanding and controlling the underlying diffusion mechanisms in charge‐storing materials remain significant challenges. Here, ultralong charge retention in epitaxially grown LaAlO_3_ (LAO) thin films is reported, enabled by anisotropic charge diffusion. The surface accumulation of oxygen vacancies, driven by the internal polar field, effectively suppresses out‐of‐plane electron hopping, allowing ≈90.9% of the initially injected charges to remain on the LAO surface after 180 h, with stable retention persisting for weeks or longer. Time‐resolved Kelvin probe force microscopy and finite‐difference simulations consistently reveal that this retention enhancement arises from diffusion anisotropy induced by surface‐localized defect states in LAO, rather than by isotropic ionic migration. These results provide an effective strategy for designing high‐performance charge storage materials based on polar‐layered oxides, paving the way for durable surface charge‐based electronic and energy devices.

## Graphical abstract

Anisotropic charge diffusion in epitaxially grown LAO thin films is reported, which enables ultralong charge retention. Surface‐accumulated oxygen vacancies effectively suppress out‐of‐plane electron hopping, allowing ≈90.9% of the surface charges to remain after a few weeks or longer. Kelvin probe force microscopy and finite‐difference simulations consistently support this mechanism for stable charge retention.

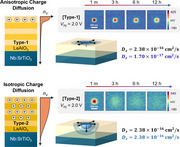

## 1 Introduction

The intrinsically limited charge transport in dielectric materials enables the long‐term retention of charges at specific locations. This charge retention, or charge storage, capability is critical for electronic devices and energy technologies that require sustained electrostatic potentials, such as charge‐trapping memory,^[^ ^1^, ^2^, ^3^ ^]^ triboelectric nanogenerators,^[^ ^4^, ^5^, ^6^ ^]^ and supercapacitors.^[^ ^7^, ^8^ ^]^ A central goal of charge storage is to stably and persistently maintain injected charges at designated locations. Various mechanisms have been explored to achieve this goal, with one of the most classical approaches relying on charge trapping in dielectric oxides, where electropositive oxygen vacancies serve as charge traps.^[^ ^9^, ^10^, ^11^ ^]^ Upon charge injection, shallow mid‐gap states associated with the point defects become occupied, and the trapped charges remain localized until released by external perturbations. However, it is widely known that such purely electronic mechanisms offer limited retention performance, as electrons can diffuse out readily via quantum tunneling.

To overcome this limitation, recent studies have adopted high‐energy electrification methods such as corona discharge, which can access deep trap states in dielectric materials and induce electret‐like behavior.^[^ ^12^, ^13^ ^]^ Despite their promise, these methods still suffer from difficulties in precisely controlling the amount of injected charge and achieving reliable electrical control over charging and discharging. As an alternative, ionic charge‐based mechanisms have been proposed. One commonly studied approach involves the direct injection of ions carrying intrinsic charges into dielectric materials.^[^ ^14^, ^15^ ^]^ Recently, employing different ions as electron donors and trapping centers, respectively, has been shown to enhance charge retention at material surfaces and interfaces.^[^ ^16^ ^]^ While recent progress in combining electronic and ionic charge strategies has further improved retention performance, note that most prior studies have mainly focused on the charge injection process. Beyond injection, the microscopic diffusion behavior of injected charges also plays an important role in charge retention, yet remains relatively unexplored.

The diffusion mechanism of injected charges strongly depends on the spatial distribution of ionic components within the dielectric, as this distribution determines the hopping pathways of electrons and governs the migration dynamics of ionic charges. In this context, polar‐layered oxides, such as the perovskite LaAlO_3_ (LAO), may serve as a promising material platform for controlling charge diffusion in a well‐defined manner. Although LAO is overall non‐polar, its intrinsic atomic stacking along the (001) direction consists of alternating atomic planes with positive and negative polarity.^[^ ^17^, ^18^, ^19^ ^]^ When LAO forms a heterostructure with a centrosymmetric, non‐polar oxide such as SrTiO_3_ (STO), a polarity discontinuity arises at the interface, inducing strong electron accumulation and forming a 2D electron gas.^[^ ^20^, ^21^, ^22^, ^23^, ^24^ ^]^ Likewise, electropositive oxygen vacancies have been reported to form readily on the LAO surface via the same polar discontinuity mechanism.^[^ ^25^, ^26^ ^]^ Because these surface oxygen vacancies originate from intrinsic potential constraints, their density and spatial distribution are highly reproducible.^[^ ^27^ ^]^ Compared to the broadly and uniformly distributed charge traps in conventional oxides, these surface‐localized oxygen vacancies in LAO are expected to induce non‐trivial and highly anisotropic charge diffusion dynamics.

In this study, we demonstrate ultralong charge retention in epitaxially grown LAO thin films, enabled by the anisotropic charge diffusion. We show that defect‐mediated electron hopping, constrained by surface‐accumulated oxygen vacancies, suppresses out‐of‐plane diffusion and enhances surface charge stability. These results establish an effective strategy for designing electronically tunable charge storage platforms based on polar‐layered oxides.

## 2 Results and Discussion

**Figure 1:**
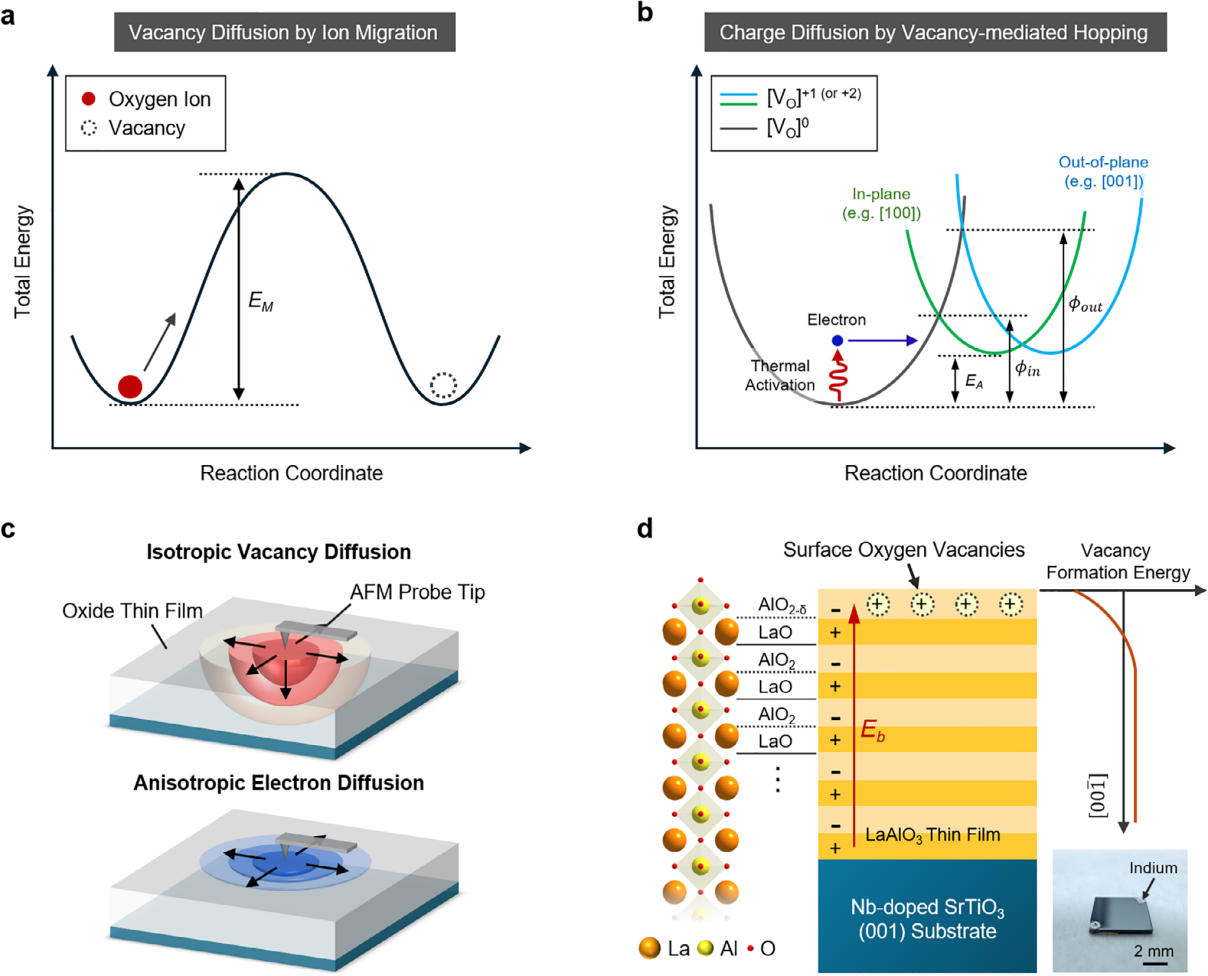
Charge diffusion mechanisms in dielectric oxides. a) Schematic coordination energy diagram describing the ionic migration process in crystalline materials. For an oxygen ion in the crystal lattice to migrate to a neighboring vacancy site, it must overcome a migration energy barrier *E_M_*. In single‐crystalline oxides, *E_M_* is generally direction‐independent, and thus this mechanism leads to isotropic charge diffusion. b) Coordination diagram describing vacancy‐mediated electron hopping. In this case, even when the activation energy *E_A_* is identical, the effective tunneling barrier can be modulated depending on the direction, leading to variation in the hopping rate. This directional dependence enables anisotropic charge diffusion. c) Schematic illustrations of isotropic vacancy diffusion (top) and anisotropic electron diffusion (bottom), induced by an atomic force microscopy probe tip on an oxide film. d) Polar‐layered LaAlO_3_ (LAO) thin film grown on TiO_2_‐terminated (001) Nb‐doped SrTiO_3_ (Nb:STO) substrate. The right panel shows the formation energy of oxygen vacancy along the *z*‐axis. The inset shows an as‐prepared standard LAO/Nb:STO sample.

The main charged species in oxides are electrons and ionized oxygen vacancy defects. When these components become locally concentrated at specific positions by a charge injection process, they naturally tend to diffuse outward over time, approaching an equilibrium distribution. In this diffusion process, electrons and oxygen vacancies move through different mechanisms. First, the diffusion of oxygen vacancies can be interpreted as the migration of oxygen ions to neighboring vacancy sites (**Figure** **1**a). This process requires energy greater than the migration energy *E_M_*,^[^ ^28^, ^29^ ^]^ which is closely related to the crystalline structure of the material (Figure S1, Supporting Information). In contrast, injected electrons in dielectric materials are typically trapped at electropositive oxygen vacancies and can move by hopping between nearby vacancy sites (Figure 1b). Hopping occurs when adjacent oxygen vacancies are positively ionized (i.e., [V_O_]^+1^ or [V_O_]^+2^), and it requires activation energy *E_A_* to overcome the energy difference between defect states (Figure S2, Supporting Information).^[^ ^30^ ^]^ Thus, both diffusion mechanisms require additional energy relative to the ground state, but, at room temperature, they can be thermally activated.

Based on these fundamental diffusion mechanisms, we focus on how to retain the accumulated charges over extended periods with minimal loss. We considered whether the migration of the charged species could be directionally controlled. In most dielectric oxides, ionic migration is generally characterized by isotropic diffusivity in both in‐plane and out‐of‐plane directions. In such cases, the oxygen vacancies are expected to diffuse simultaneously in all three dimensions (the top panel of Figure 1c). In contrast, the diffusion of electrons can be made anisotropic by controlling the spatial distribution of oxygen vacancy defects, which are electron‐trapping sites (the bottom panel of Figure 1c). For example, in a system where oxygen vacancies are confined in a 2D in‐plane distribution, electrons should tunnel a greater distance in the out‐of‐plane direction compared to the in‐plane direction to diffuse out (Figure S3, Supporting Information). In this case, the *E_A_* for hopping remains the same in both directions, but the effective heights of the tunneling barriers (see ϕ_*in*_ and ϕ_*out*_ in Figure 1b) differ by direction. This directional asymmetry helps suppress out‐of‐plane charge loss and improves overall retention performance.

To verify this hypothetical charge diffusion anisotropy, we chose LAO thin films epitaxially grown on 0.5% Nb‐doped SrTiO_3_ (001) (Nb:STO) substrates as a model system (Figure 1d). A 20‐nm‐thick single‐crystalline LAO film was synthesized on a TiO_2_‐terminated Nb:STO substrate by pulsed laser deposition (see Experimental section for details). The as‐grown LAO film is highly insulating, whereas the Nb:STO substrate serves as a conductive bottom electrode. In LAO/Nb:STO heterostructures, previous studies have shown that the formation energy of oxygen vacancies is relatively lower at the top surface than in the interior, due to the internal built‐in field (right panel of Figure 1d). As a result, a specific amount of oxygen vacancies remains at the LAO surface,^[^ ^26^, ^27^ ^]^ even when the film is cooled slowly in an oxygen‐rich atmosphere after deposition. This surface‐accumulated oxygen vacancies are expected to allow charges injected at the LAO surface to diffuse preferentially in‐plane, while out‐of‐plane diffusion is strongly suppressed.

**Figure 2:**
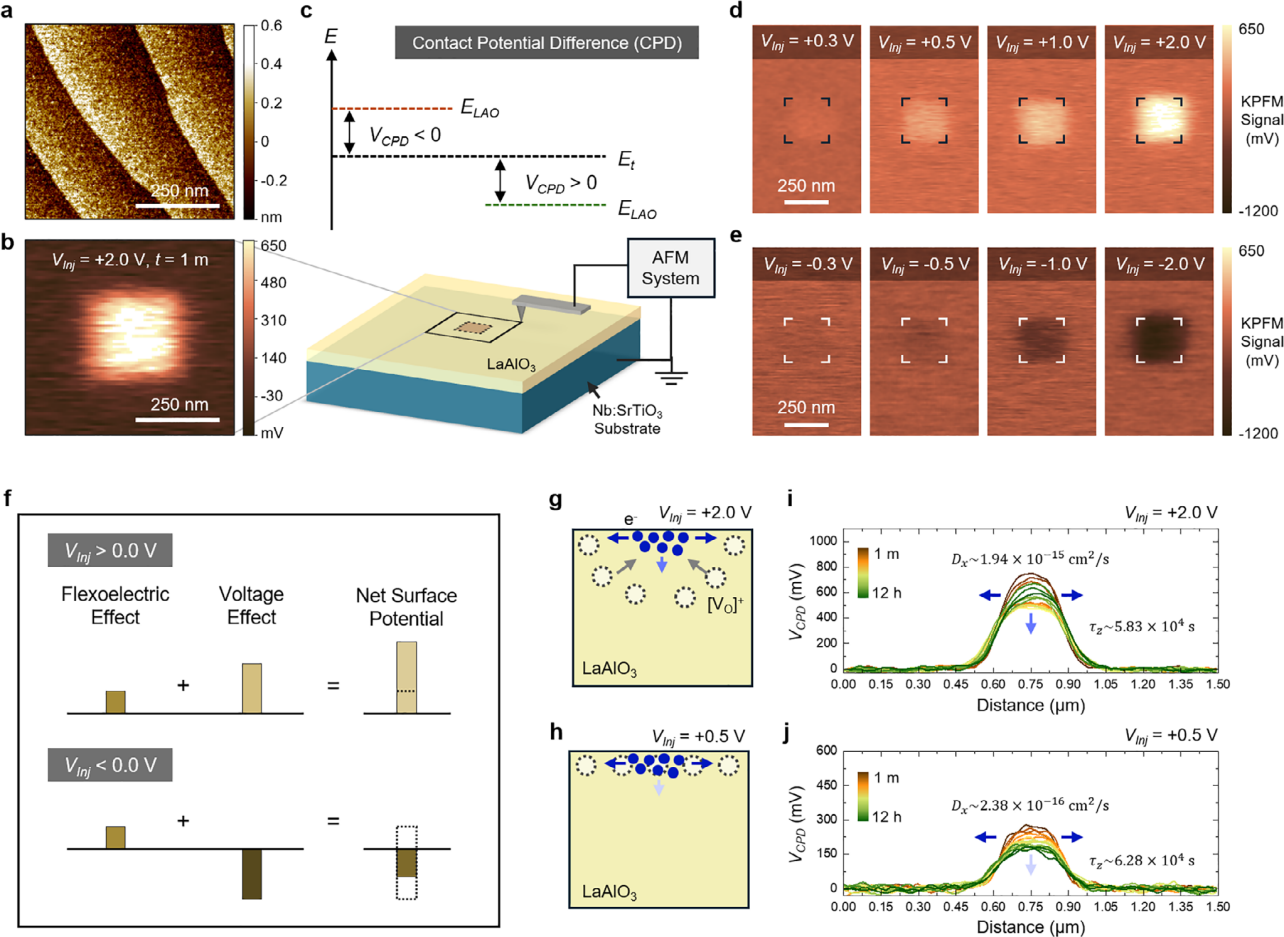
Charge injection and diffusion characteristics of stoichiometric LAO thin films, probed by the Kelvin probe force microscopy (KPFM) technique. a) Surface topography image of an as‐grown LAO film obtained by atomic force microscopy (AFM). b) KPFM image measured right after the charge injection process. The bright‐colored region at the center shows the charge‐injected zone. c) Schematic illustration of the contact potential difference (CPD) *V_CPD_*. d,e) KPFM images showing the surface potential change by the charge injection with different d) positive and e) negative tip bias voltages *V_Inj_*. f) Schematic illustration of the asymmetric surface potential response under opposite tip biases. Negative *V_Inj_* lowers the surface potential, while positive *V_Inj_* raises it. Regardless of the bias polarity, however, the flexoelectric response generates a positive surface potential due to tip‐induced strain. The combination of charge polarity and unidirectional flexoelectric signal results in asymmetric CPD shifts. g) As the *V_Inj_* is as high as +2.0 V, both ionic and electronic distributions are locally changed, resulting in a combination of ionic and electronic diffusions. h) In contrast, as the *V_Inj_* is as small as +0.5 V, the ionic distribution is not changed, leading to only the electronic diffusion. i,j) Time‐dependent *V_CPD_* profiles acquired at 1 h intervals over 12 h for *V_Inj_* of i) +2.0 V and j) +0.5 V. The in‐plane diffusivity *D_x_* and the out‐of‐plane decay time constant *τ_z_* are indicated inside plots. The blue arrows indicate the diffusion directions.

We investigate the charge diffusion behaviors on LAO thin films using atomic force microscopy (AFM) and a Kelvin probe force microscopy (KPFM) system. **Figure** **2**a shows an AFM topography image of an as‐grown LAO/Nb:STO heterostructure. The well‐defined step‐and‐terrace structure confirms the high quality of the epitaxially grown LAO thin films. On the atomically flat and clean LAO surface, we injected charge into a square region by scanning a 200 nm × 200 nm area using a conductive AFM probe tip with a constant bias voltage *V_Inj_*. Figure 2b shows a KPFM image measured immediately after charge injection with *V_Inj_* of +2.0 V. The surface potential within the central square region is clearly higher than that of the surrounding area, indicating successful charge injection. Given that the surface topography was not changed by the charge injection process (Figure S4, Supporting Information), we confirm that this change of surface potential is not attributed to external factors such as surface adsorbates or bulk defects. Figure 2c schematically illustrates how we interpret the measured KPFM signals from the LAO sample. The KPFM measures the contact potential difference (*V_CPD_*) between the AFM tip and the LAO surface. Thus, assuming a constant energy level of the probe tip (*E_t_*) in a well‐controlled experimental environment, the *V_CPD_* value directly captures the energy level of the LAO surface (*E_LAO_*). As shown in Figure 2b, the positive *V_Inj_* resulted in the positive *V_CPD_*, which means the *E_LAO_* decreased. This indicates that the positive *V_Inj_* injected negative charges onto the LAO surface, which is quite reasonable. It should be noted that, however, the injected negative charges may originate either from an increased local electron density or from a decreased concentration of oxygen vacancies. Therefore, a further analysis is required to distinguish the effects of electronic charges from those of ionic charges, as will be revisited in the later part.

Figure 2d,e presents KPFM images measured after charge injection with different *V_Inj_*. It is evident that both negative and positive charges can be effectively injected into LAO, depending on the polarity of *V_Inj_*, and that the amount of injected charge increases proportionally with *V_Inj_*. Before going further, we note that the effects of charge injection under positive and negative *V_Inj_* are not symmetric. Even when the magnitude of *V_Inj_* is the same, the change in the KPFM signal is more pronounced under positive bias than under negative bias. This asymmetry is attributed to flexoelectricity, a phenomenon that has recently attracted increasing attention.^[^ ^31^, ^32^, ^33^ ^]^ When a localized mechanical force is applied to an oxide surface, a stress‐induced flexoelectric field is generated in the surrounding region, which can alter the distribution of oxygen vacancies without applying an electric field.^[^ ^34^ ^]^ In other words, during the charge injection process, a small amount of oxygen vacancies inevitably migrate outward in the same direction regardless of the polarity of *V_Inj_* (Figure 2f), leading to a greater amount of charge being injected under positive *V_Inj_*. Therefore, unless otherwise noted, we focus on the case of positive *V_Inj_* and a relatively gentle contact force of 30 nN in the following discussions. The influence of the flexoelectric effect on charge retention behavior is examined in more detail in Figure S5 (Supporting Information).

Let us now return to the earlier point that the injected charge can be either electronic or ionic. The influence of these charged species can be controlled by the magnitude of *V_Inj_*. For example, if *V_Inj_* is sufficiently large to induce oxygen vacancy migration during the charge injection process, the subsequent diffusion process will inevitably involve not only electron diffusion but also oxygen ionic migration (Figure 2g). In contrast, when *V_Inj_* is sufficiently small and the distribution of oxygen vacancies remains nearly unchanged during the charge injection, the following diffusion behavior is dominated by electron dynamics alone (Figure 2h). From the Nernst–Planck framework, we assume that when charge injection is performed with *V_Inj_* ≤ +2.5 V, the ionic contribution remains minor (Note S1, Supporting Information).

Figure 2i,g shows *V_CPD_* line profiles extracted from KPFM images measured at 1 h intervals over a 12 h period, illustrating how the square‐patterned charges injected with *V_Inj_* = +2.0 V and *V_Inj_* = +0.5 V, respectively, diffuse over time. The time‐dependent diffusion behavior has often been analyzed by Fick's law.^[^ ^35^, ^36^, ^37^ ^]^ However, since the KPFM signals do not directly capture the buried charges in films, the out‐of‐plane diffusivity cannot be accurately estimated using a conventional Fick's 2nd law equation. Therefore, we employ a modified Fick's law model, represented by a diffusion equation as below.

(1) $$\frac{\partial C\left(x,t\right)}{\partial t}=D_{x}\frac{\partial^{2}C\left(x,t\right)}{\partial t^{2}}-\frac{C\left(x,t\right)}{\tau_{z}}$$

C (*x*, *t*) is the time‐dependent spatial distribution of surface charge. *D_x_* and *τ_z_* represent the in‐plane diffusivity and the out‐of‐plane decay time constant, respectively. Given the symmetric crystalline structure of LAO, we assume that the diffusivities along the *x*‐ and *y*‐directions are identical in the in‐plane configuration. Using this modified diffusion equation, we extracted values of *D_x_* ≈ 1.94 × 10^−15^ cm^2^ sec^−1^ and τ_*z*_ ≈ 5.83 × 10^4^ sec from the data set obtained with *V_Inj_* of +2.0 V. In contrast, when the charges were injected by a smaller *V_Inj_* of +0.5 V, *D_x_* ​ decreased to ≈2.38 × 10^−16^ cm^2^ sec^−1^, and *τ_z_* increased to ≈6.28 × 10^4^ sec. These results indicate that when the *V_Inj_* is as small as +0.5 V, where electron diffusion is dominant, charge diffusion is significantly suppressed. This is rather surprising, as electron diffusion is generally considered to be relatively fast in oxygen‐deficient oxides.

**Figure 3:**
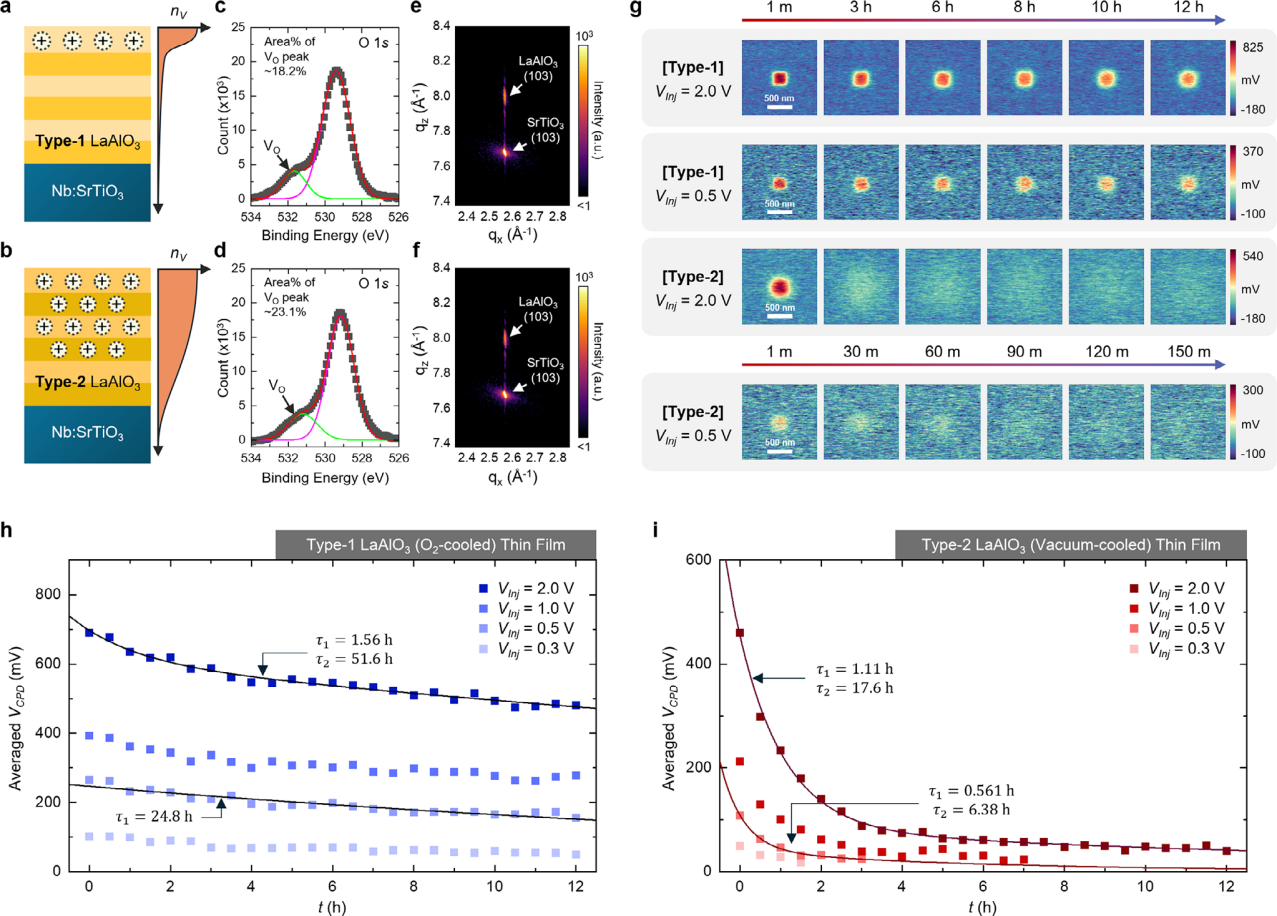
Anisotropic charge diffusion enabled by the surface‐accumulated oxygen vacancies. a,b) Schematic illustrations of oxygen vacancy distributions in a) a standard (Type‐1) and b) oxygen‐deficient (Type‐2) LAO films. The right‐side plots show the expected distributions of oxygen vacancies (*n_V_*). c,d) XPS O 1*s* core‐level spectra of the c) Type‐1 and d) Type‐2 samples. The peaks centered at 529.4 eV (pink) and at 531.7 eV (green) correspond to lattic oxygen and oxygen vacancies, respectively. The red line represents the overall fitting curve, and the black squares indicate the measured data. e,f) XRD reciprocal space maps of the (103) reflections for the e) Type‐1 and f) Type‐2 samples. g) Time‐dependent evolution of the surface potential in the Type‐1 and Type‐2 samples. Representative data sets corresponding to charge injection under *V_Inj_* = +2.0 and +0.5 V were selected for comparison. h,i) Time‐dependent evolution of the averaged *V_CPD_* within the central 25 × 25 pixels of the charge‐injected region in h) Type‐1 and i) Type‐2 samples. The black lines represent fitting curves, and the extracted time constants are indicated.

To explore the origin of the exceptionally slow electron diffusion observed at the LAO surface, we prepared two types of samples with different oxygen vacancy distributions and comparatively analyzed their diffusion behaviors. **Figure** **3**a,b illustrates schematic representations of oxygen vacancy distributions in a standard (Type‐1) and an oxygen‐deficient (Type‐2) LAO/Nb:STO heterostructure, respectively. Both types of LAO films were grown under the same conditions. However, the Type‐1 LAO film was slowly cooled to room temperature in an oxygen‐rich atmosphere after deposition to minimize the number of oxygen vacancies. As previously discussed, under such oxidizing conditions, oxygen vacancies accumulate near the film surface to compensate for the internal polar field. In contrast, the Type‐2 film was cooled in a vacuum (oxygen partial pressure ≈7 × 10^−7^ Torr) after deposition. This cooling process leads to a significantly higher concentration of oxygen vacancies, which are expected to be more uniformly distributed throughout the film.

The difference in oxygen deficiency between the two samples was verified by X‐ray photoelectron spectroscopy (XPS). Figure 3c,d presents the O 1*s* core‐level XPS spectra obtained from the Type‐1 and Type‐2 samples, respectively. The oxygen vacancy‐related peak centered at 531.7 eV^[^ ^38^ ^]^ accounted for ≈18.2% of the total O 1*s* peak area in the Type‐1 sample, whereas it increased to ≈23.1% in the Type‐2 sample. This result indicates that, as intended, a higher concentration of oxygen vacancies was formed in the Type‐2 sample. We also verified through XPS analyses of the La 3*d_5/2_* and Al 2*p* core‐level spectra that these oxygen deficiencies in both samples are not severe enough to alter the cation stoichiometry of LAO (Figure S6, Supporting Information). Additionally, since excessive oxygen deficiency could degrade the crystallinity of LAO films and introduce extrinsic effects into the charge retention characteristics, we performed X‐ray diffraction (XRD) analysis to ensure that both LAO films possesse similarly high crystalline quality. Figure 2e,f shows the reciprocal space mapping (RSM) results obtained from each sample. These RSM data clearly demonstrate that both LAO films were coherently grown on the Nb:STO (001) substrates, and their crystallinity was not compromised by oxygen deficiency. The corresponding XRD theta‐2theta patterns are given in Figure S7 (Supporting Information).

Figure 3g shows the time‐dependent charge diffusion characteristics of the Type‐1 and Type‐2 samples, visualized by KPFM under different *V_Inj_*. To investigate the voltage dependence of charge diffusion, we performed long‐term KPFM measurements with two different *V_Inj_*: +2.0 and +0.5 V. When *V_Inj_* exceeds ≈+3.0 V, an unusual behavior was observed in which the surface potential initially increases but subsequently decreases (Figure S8, Supporting Information). This unexpected phenomenon is likely caused by the adsorption of ambient molecules onto the LAO surface,^[^ ^39^ ^]^ induced by the strong electric field around the conductive AFM probe tip. Therefore, such high voltages are considered unsuitable for observing intrinsic charge retention characteristics. We verified that no such extrinsic effects occur up to *V_inj_* = +2.5 V (Figure S9, Supporting Information). It should be noted, however, that the threshold voltage for triggering these extrinsic effects may vary with the specific material system and environmental perturbations. Note that the Type‐1 sample exhibits stable charge retention for up to 12 h under both *V_Inj_* conditions, whereas the Type‐2 sample shows significantly faster charge diffusion. In particular, for the Type‐2 sample, the surface charge injected by *V_Inj_* of +0.5 V diffused rapidly, and no meaningful signal was detected just after 90 min (see the bottom panel of Figure 3g). The fact that the Type‐2 sample, despite having a relatively larger number of oxygen vacancies, exhibited faster charge diffusion indicates that oxygen vacancy‐mediated electron hopping plays an important role in charge retention, as we hypothesized. Additionally, both types of samples exhibit a two‐step diffusion behavior: a relatively rapid charge diffusion within the first 2–3 h, followed by a slower diffusion at longer times.

To quantify the two‐step diffusion behavior, we fitted the time‐dependent averaged surface potential, measured within the square patterns, using exponential decay functions (Figure 3h,i). In the Type‐1 sample, the decay of surface potential following charge injection under a small *V_Inj_* of +0.5 V is well described by a typical single‐exponential function. Since a low *V_Inj_* of +0.5 V is insufficient to induce significant ionic migration, this single‐exponential decay can be considered mainly as an electronic process. In contrast, when a higher *V_Inj_* of +2.0 V was used, the decay exhibits a biexponential behavior, which can be expressed by the following equation (Equation (2)):

(2) $$P_{surface}\left(t\right)=P_{0}+A_{1}e^{-\frac{t}{\tau_{1}}}+A_{2}e^{-\frac{t}{\tau_{2}}}$$

As indicated in the plot, the two extracted time constants differ substantially (τ_1_ ≈ 1.56 h and τ_2_ ≈ 51.6 h), suggesting the coexistence of two distinct charge diffusion processes with different timescales. Based on the results at +0.5 V, we can attribute the slower component (*τ_2_* ​) to electronic diffusion, while the faster component (*τ_1_* ​) is associated with ionic diffusion. In the Type‐2 sample, a similar biexponential decay behavior was observed, with both time constants being significantly smaller than those of the Type‐1 sample. This indicates that both electronic and ionic diffusion processes occur more rapidly in the Type‐2 sample. These analyses provide two important insights. First, although both electronic behavior and ionic processes are involved in charge decay, the exceptionally slow decay in the Type‐1 sample is predominantly attributed to electronic behavior. Second, as confirmed by the temporal evolution of KPFM images, confining oxygen vacancies near the surface effectively suppresses out‐of‐plane electron diffusion and is therefore advantageous for long‐term charge retention.

**Figure 4:**
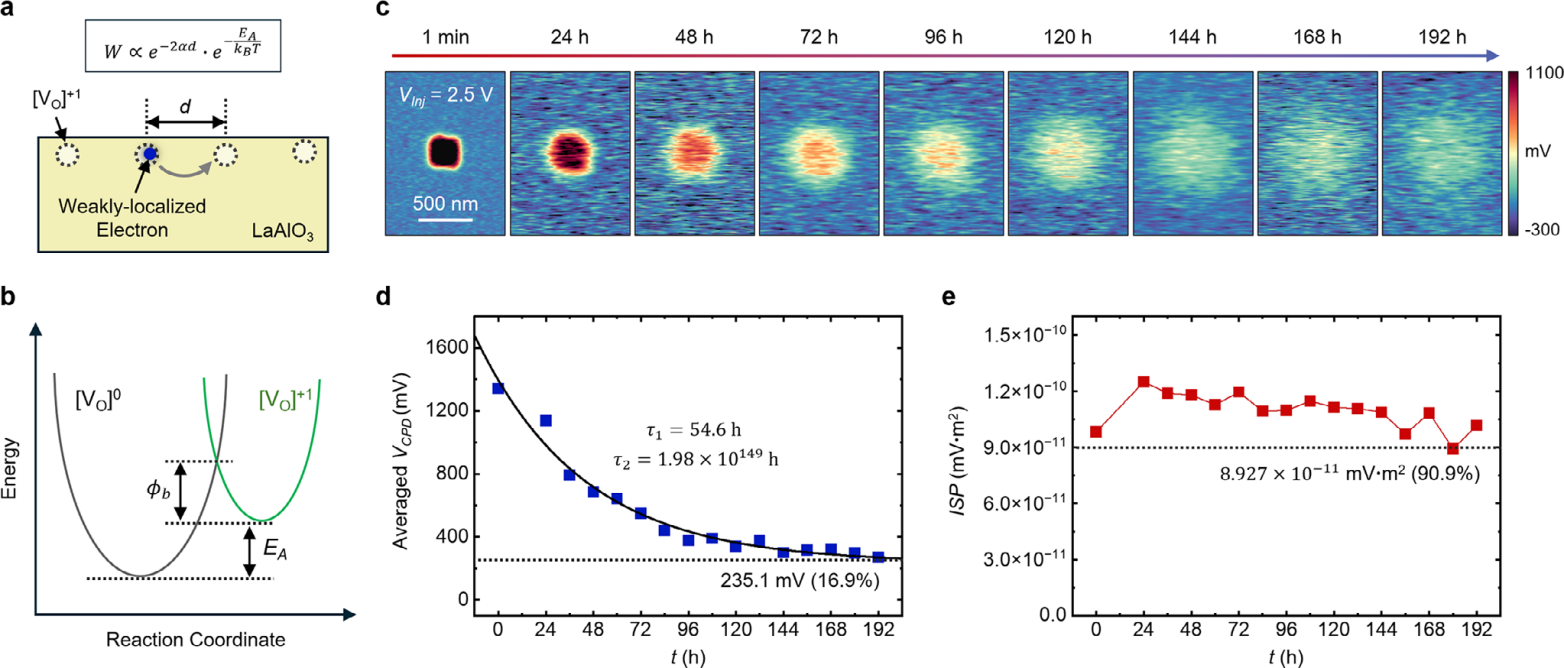
Ultralong charge retention in Type‐1 LAO/Nb:STO heterostructure. a) Schematic depiction of electron hopping between oxygen vacancy cites separated by a distance of *d*. b) Coordination diagram describing electron hopping between oxygen vacancy cites. *E_A_* and ϕ_*b*_ denote the activation energy for hopping and the effective tunneling barrier height, respectively. c) Long‐term monitoring of the injected charges in the Type‐1 sample using KPFM. Charge injection was performed on the LAO surface by *V_Inj_* of +2.5 V. d) Time‐dependent evolution of *V_CPD_* values averaged within the central 25 × 25 pixels of the charge‐injected region. The black curve represents a biexponential fitting with extracted time constants. Note that the *V_CPD_* value stabilized at ≈16.9% of the initial value after one week. e) Time‐dependent integrated surface potential (ISP). At 180 h, where the *ISP* reached its minimum value, it retained 90.9% of the initial value and 71.4% of the maximum value observed at 24 h.

This anisotropic charge diffusion can be rationalized by the Miller–Abrahams model,^[^ ^40^, ^41^ ^]^ which describes how hopping conduction of weakly localized electrons is governed by energy and spatial factors. According to the Miller–Abrahams hopping rate equation, the probability that a localized electron hops to a neighboring defect state that is slightly higher in energy by *ΔE* (**Figure** **4**a) is given by the equation (Equation (3)):

(3) $$\Gamma \propto e^{-2\alpha d}\cdot e^{-\frac{\Delta E}{k_{B}T}}$$

Γ, α, *d*, *k_B_*, and *T* represent the hopping rate, the inverse localization length, the distance between two oxygen vacancies, the Boltzmann constant, and temperature, respectively. Given the constant energy difference *ΔE* between oxygen vacancy defect states in single‐crystalline LAO systems,^[^ ^42^ ^]^ note that the hopping rate is mainly determined by the averaged distance *d* between surface oxygen vacancies (Figure 4b). Therefore, the charge retention performance can be maximized when 1) the number of charge‐trapping sites is sufficient to capture a significant amount of externally injected charge, but 2) not so high that hopping becomes overly facile due to the small *d*. In this regard, the Type‐1 sample satisfies both conditions, allowing the injected charge to remain on the surface for a long period. For clarity, all variables and parameters used in the modeling are summarized in Table S1 (Supporting Information).

Figure 4c presents the long‐term monitoring results of the injected charges in the Type‐1 sample, clearly demonstrating its remarkable charge retention characteristics. In the initial stage, where both ionic migration and electronic hopping coexist, a relatively rapid charge loss was observed. However, after ≈96 h, charge diffusion became negligible, and the surface charge stabilized at ≈16.9%, with no further decrease observed thereafter (Figure 4d). The long‐term decay of the surface potential can also be accurately fitted with a biexponential function. Notably, one of the extracted time constants is unphysically large (τ_2_ ≈ 1.98 × 10^149^ h), indicating that the surface charge configuration reaches a metastable equilibrium state ≈96 h after charge injection.

While the evolution of *V_CPD_* value effectively captures the decaying behavior of the surface potential due to charge diffusion, it is not suitable for quantitatively assessing the overall loss of surface charge. We instead introduce the Integrated Surface Potential (ISP), which accounts for in‐plane diffusion effects and thus provides a more representative metric for tracking the temporal evolution of overall surface charge. Assuming that the *V_CPD_* value is uniform within each pixel area (≈7.8 nm  × ≈7.8 nm), the ISP was calculated by summing the product of the *ΔV_CPD_* values (i.e., the change in *V_CPD_* induced by charge injection process at each pixel in the KPFM map) and the corresponding pixel area (the detailed method is explained in Note S2, Supporting Information). Figure 4e shows the time‐dependent evolution of the ISP value. Remarkably, even after 180 h, the ISP retained over 90.9% of its initial value. This indicates that, despite a discernible decrease in local surface potential due to in‐plane diffusion (Figure 4d), the majority of the injected charges remained on the surface for more than 180 h. We further extended the monitoring of the surface potential and ISP using the same sample, and found that the surface charges remained stably preserved even after several weeks, as confirmed by KPFM images and ISP values (Figure S10, Supporting Information).

To further validate the effect of the anisotropic charge diffusion on charge retention properties, we performed finite‐difference simulations of time‐dependent charge diffusion in LAO thin films. Unlike KPFM analyses, these simulations can directly apply Fick's second law without modification, enabling an accurate description of the 3D charge diffusion dynamics. In the simulation, the conceptual LAO system is modeled as a thin slab with dimensions of 1.5 µm × 1.5 µm × 20 nm. Charges were initially introduced in a localized square region (30 × 30 grids, corresponding to 23.4 nm × 23.4 nm) at the center of the surface with 1000 arbitrary‐unit charges per grid, and then the time evolution of the charge distribution was calculated using an explicit finite‐difference scheme. The in‐plane charge diffusivity value was consistently set to be *D_x_* ≈ 2.38 × 10^−16^ cm^2^ sec^−1^, which was estimated from the experimental results. To implement charge loss, we employed an absorbing boundary condition at the bottom of the film, assuming that charges diffused beyond the film thickness were irreversibly lost through the conductive Nb:STO. The detailed simulation method is described in Note S3 (Supporting Information).

**Figure 5:**
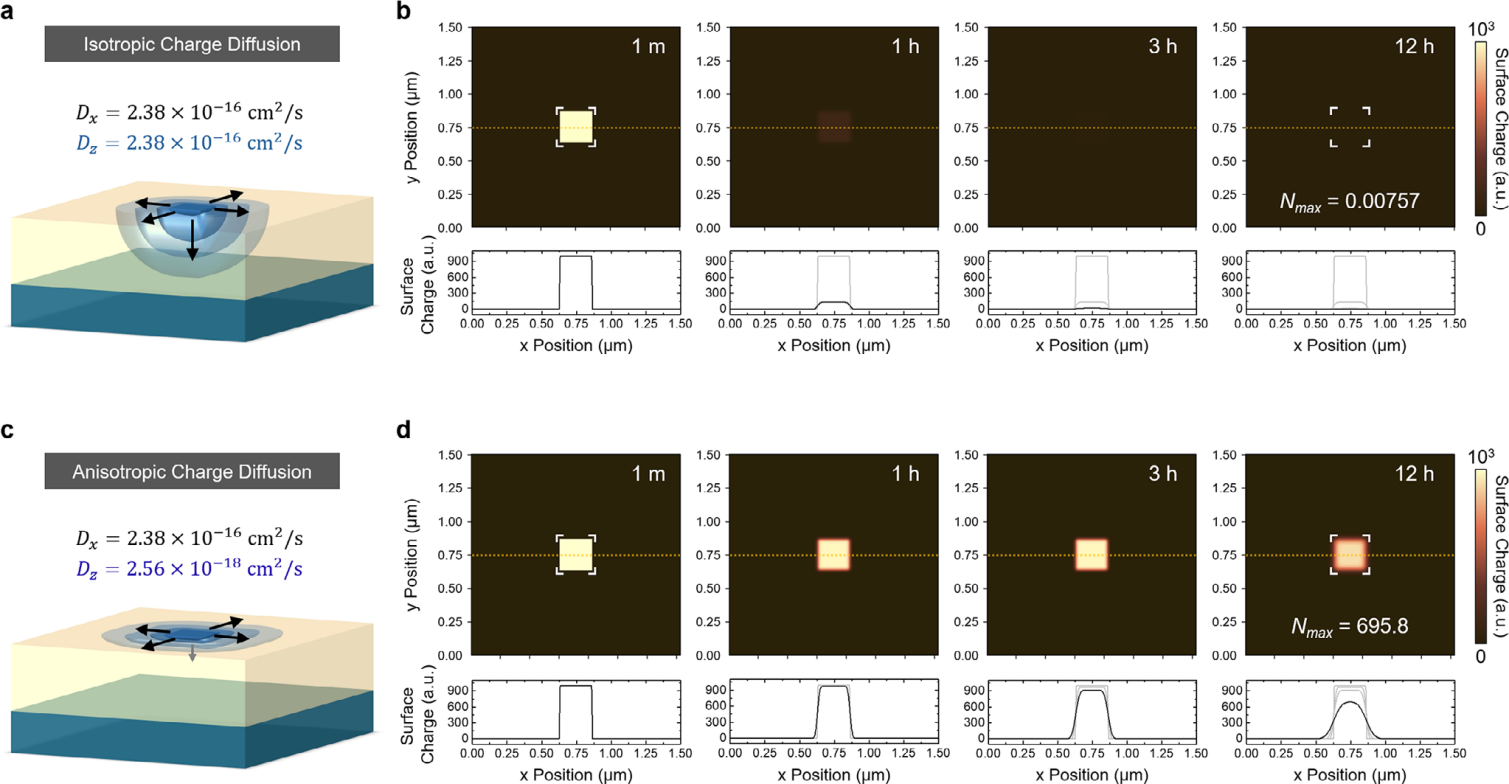
Simulation of isotropic and anisotropic charge diffusion in LAO thin films. a) Schematic illustrating isotropic charge diffusion. Both the in‐plane and out‐of‐plane diffusivities are set to be *D_x_* = *D_z_*  =  2.38 × 10^−16^cm^2^ sec^−1^. b) Simulated surface charge distributions at 1 min, 1, 3, and 12 h after the conceptual charge injection. Below each image, line profiles along the yellow dashed line (*y* = 0.75 µm) show the surface charge density as a function of *x*‐position. c) Schematic illustrating anisotropic charge diffusion. In this case, *D_z_* was set to be 2.56 × 10^−18^cm^2^ sec^−1^. d) Simulated results showing significantly improved surface charge retention. At 12 h, 695.8 arbitrary‐unit charges (i.e., 69.6% of the initial value) remain at the center, closely matching the experimental trend observed in the Type‐1 sample.

We first simulated isotropic charge diffusion in LAO (**Figure** **5**a), assuming that the out‐of‐plane diffusivity *D_z_* was equal to *D_x_* (i.e., *D_z_* = *D_x_*  =  2.38 × 10^−16^ cm^2^ sec^−1^). Figure 5b shows the evolution of the surface charge distribution over time. The numerical charge profiles are plotted below each simulated image for clarity. Notably, after only 1 h, the central region retained ≈130 charges per grid, indicating that most of the initially injected charges had diffused away. This rapid charge loss closely resembles the decay behavior observed in the Type‐2 sample (Figure 3g). Since the remnant surface charge became too small to discern clearly after 1 h, a contrast‐rescaled image is provided in Figure S11 (Supporting Information) for better visualization.

We next reduced *D_z_* to simulate anisotropic charge diffusion (Figure 5c). A series of simulations was performed using various values of *D_z_*, and we found that when *D_z_* ​ was ≈1.1% of *D_x_* (i.e., *D_z_* =  2.56 × 10^−18^ cm^2^ sec^−1^), the simulated diffusion behavior closely matched the experimental observations. As shown in Figure 5d, surface charge was retained significantly longer, and ≈69.6% of the initially injected charges remained on the surface after 12 h, in good agreement with the experimental data (Figure 3h). Additional results for surface potential evolution under different anisotropy conditions are presented in Figure S12 (Supporting Information). The simulation parameters and the corresponding relative residual charge (%) are directly compared with the experimentally observed values in Table S2 (Supporting Information). These finite‐difference simulation results strongly support our conclusion that anisotropic charge diffusion plays a critical role in enhancing surface charge retention. Importantly, it is apparent that the surface‐localized oxygen vacancies in LAO thin films are key to enabling this anisotropic charge diffusion.

## 3 Conclusion

In summary, we have demonstrated that single‐crystalline LAO thin films grown on Nb:STO, where oxygen vacancies are inherently accumulated near the surface due to the internal polar field, exhibit highly anisotropic charge diffusion, thereby enabling significantly enhanced long‐term charge retention. Our KPFM analyses and finite‐difference simulations, consistently confirm that anisotropic charge diffusion plays a crucial role in stabilizing surface‐injected charges in LAO. Thus, anisotropic charge diffusion can serve as a powerful design principle for functional materials targeting ultralong charge retention. For comparison, the charge retention properties observed in LAO thin films are summarized in Table S3 (Supporting Information) alongside those of previously reported oxide systems. Beyond LAO, most insulating polar‐layered oxides can also accumulate oxygen vacancies at their surfaces or interfaces through similar mechanisms, making them suitable for achieving ultralong charge retention. Moreover, even in non‐polar‐layered oxides, if oxygen vacancies can be confined within quasi‐2D planes, the directional charge diffusion is expected to be attainable. Ultimately, combining the proposed engineering of directional charge diffusion with state‐of‐the‐art charge injection methodologies will provide a comprehensive strategy for significantly enhancing charge retention across a wide range of dielectric materials. Such rationally designed high‐performance charge storage materials can improve the reliability of charge‐trapping memory devices and serve in diverse applications that require a stable electrostatic field, including triboelectric nanogenerators and supercapacitors. Along with the practical implications, the ability to understand and control anisotropic charge diffusion may provide valuable insights into various fundamental phenomena, including crystal‐orientation‐dependent polaronic conduction,^[^ ^43^ ^]^ nematic transport in Ruddlesden–Popper oxides,^[^ ^44^ ^]^ and quantum transport near grain boundaries in polycrystalline systems.^[^ ^45^ ^]^

## 4 Experimental Section

### Thin Film Deposition

A pulsed laser deposition (PLD) with a KrF excimer laser (λ = 248 nm) was used to synthesize the single‐crystalline LAO thin films on Nb:STO substrates. All LAO thin films were directly grown on 0.5 wt.% Nb‐doped STO (001) substrates (CrysTec). Before film growth, the Nb:STO substrates were etched by buffered‐HF for 1 min and then annealed at 950 °C for 4 h. Subsequently, a LAO target was ablated by the excimer laser with a repetition rate of 3 Hz to grow LAO thin films. During film growth, the substrate temperature and the oxygen partial pressure were kept as 550 °C and 7.5 × 10^−4^ Torr, respectively. After film growth, Type‐1 samples were cooled in an oxygen atmosphere of 1 atm, while Type‐2 samples were cooled in the same oxygen pressure of 7.5 × 10^−4^ Torr. The film thickness of all the samples was kept as 50 unit cells (≈20 nm), which were confirmed by the X‐ray reflectometer.

### KPFM Measurements

The surface potential before and after charge injection was measured using the Kelvin probe force microscopy (KPFM) mode of an atomic force microscopy (AFM) system (NX10, Park Systems). The frequency modulated‐KPFM was performed with the AC modulation voltage of 1.0 V at 17 kHz. For the KPFM measurements, Cr/Pt‐coated tips (ElectriMulti75‐G, Budget) were used. All measurements were performed under a well‐controlled laboratory conditions: Temperature of 24 ± 2 °C and relative humidity of 25–35%.

### Finite‐Difference Simulations

Finite‐difference simulations were performed in MATLAB based on Fick's second law over a 3D domain (1.5 µm × 1.5 µm × 20 nm), discretized into 192 × 192 × 3 grid points. This grid size was chosen to match the pixel resolution of the experimental KPFM images. The diffusion of the arbitrary‐unit charges was simulated using various in‐plane (*D_x_*) and out‐of‐plane (*D_z_*) diffusivities, and the time step was determined by the stability condition of the explicit finite‐difference scheme. To emulate charge loss, an absorbing boundary condition was applied at the bottom surface (*z* = 4), and the initial charge was injected in a 30 × 30 grid region at the center of the LAO surface.

## Conflict of Interest

The authors declare no conflict of interest.

## Supporting information



Supporting Information

## Data Availability

The data that support the findings of this study are available from the corresponding author upon reasonable request.
