# Supplementary material for: Anisotropic Charge Diffusion in Polar‐Layered Oxides for Ultralong Charge Retention
Source: Adv Sci (Weinh). 2025 Oct 8;13(2):e14554. doi: 10.1002/advs.202514554 (PMC12786369; doi:10.1002/advs.202514554)
Supplement: Supplementary file 1 — Supporting Information [file ADVS-13-e14554-s001.docx]

Supporting Information

Anisotropic Charge Diffusion in Polar-layered Oxides for Ultralong Charge Retention

Sungjun Choi, Yujin Choi, Jeongdae Seo, Sanghyeok Ryou, Minwoo Jang, Haeyun Song, Sanghoon Yeom, and Hyungwoo Lee*

*Corresponding author E-mail: [hyungwoo@ajou.ac.kr](mailto:hyungwoo@ajou.ac.kr)

**Contents**

**Figure S1.** Ionic migration and defect-mediated electron hopping in crystals.

**Figure S2.** Electron hopping between oxygen vacancy defects in LaAlO_3_ (LAO).

**Figure S3.** Tunneling barrier height and activation energy for electron hopping depending on oxygen deficiency in LAO thin film.

**Figure S4.** Surface topography before and after charge injection.

**Figure S5.** Surface potential change induced by mechanical contact only.

**Figure S6.** Cation stoichiometry analyses by X-ray photoelectron spectroscopy (XPS).

**Figure S7.** Structural analyses of LAO/Nb:STO heterostructures by X-ray diffraction (XRD).

**Figure S8.** Time-dependent evolution of the averaged KPFM signals obtained from the Type-1 sample with different *V_Inj_*.

**Figure S9.** Charge retention characteristics in an LAO thin film over an extended period.

**Figure S10.** Simulated images of remnant surface charges at several hours after charge injection.

**Figure S11.** Charge retention under different diffusion anisotropies.

**Figure S12.** Material structure and key parameters for simulation of charge diffusion.

**Supplementary Note S1.** Electronic and ionic contributions to charge retention.

**Supplementary Note S2.** Integrated surface potential (ISP) calculation.

**Supplementary Note S3.** Finite-difference simulations of charge diffusion in LAO thin films.

**Supplementary Table S1.** Summary of variables and parameters

**Supplementary Table S2.** Summary of simulation parameters and results

**Supplementary Table S3.** Charge retention properties of various oxide systems


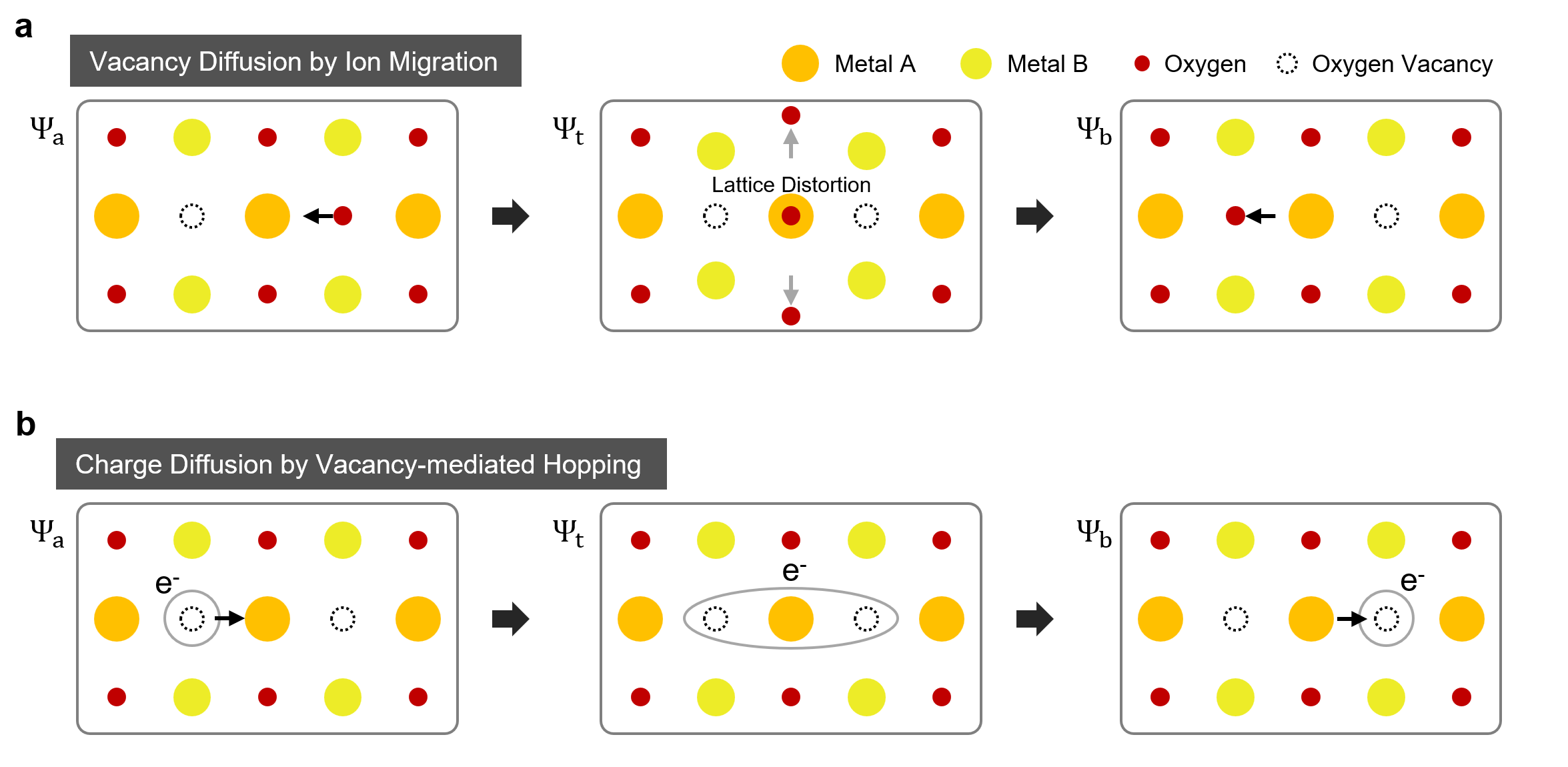


**Figure S1.** Ionic migration and defect-mediated electron hopping in crystals. **(a)** Schematic illustration of vacancy diffusion in LaAlO_3_ (LAO). Vacancy diffusion is equivalent to the migration of oxygen ions in the opposite direction. The total energy of the system remains unchanged between the initial ($\Psi_{a}$) and the final ($\Psi_{b}$) states of the oxygen ion. However, as shown in the schematic, oxygen ion migration within the perovskite lattice requires passing through a transition state ($\Psi_{t}$), which inevitably induces lattice distortion due to Coulomb interactions. The minimum energy required to overcome this structural change and enable ionic migration is known as the migration energy (*E_M_*), as shown in Figure 1a. Apparently, the *E_M_* is directly dependent on the crystalline structure of the material system. On most oxide surfaces, however, *E_M_* is comparable or smaller than the thermal energy at room temperature, making vacancy diffusion a time-dependent process that occurs naturally under ambient conditions. **(b)** Another charge diffusion mechanism is vacancy-mediated electron hopping, in which oxygen vacancies act as hopping sites. In this process, the electron wavefunction overlaps with adjacent vacancy sites, enabling electron hopping between them. This mechanism resembles a quantum tunneling process across an energy barrier. Accordingly, both the distance between vacancy sites and the height of the energy barrier within the lattice play critical roles in determining the hopping probability.


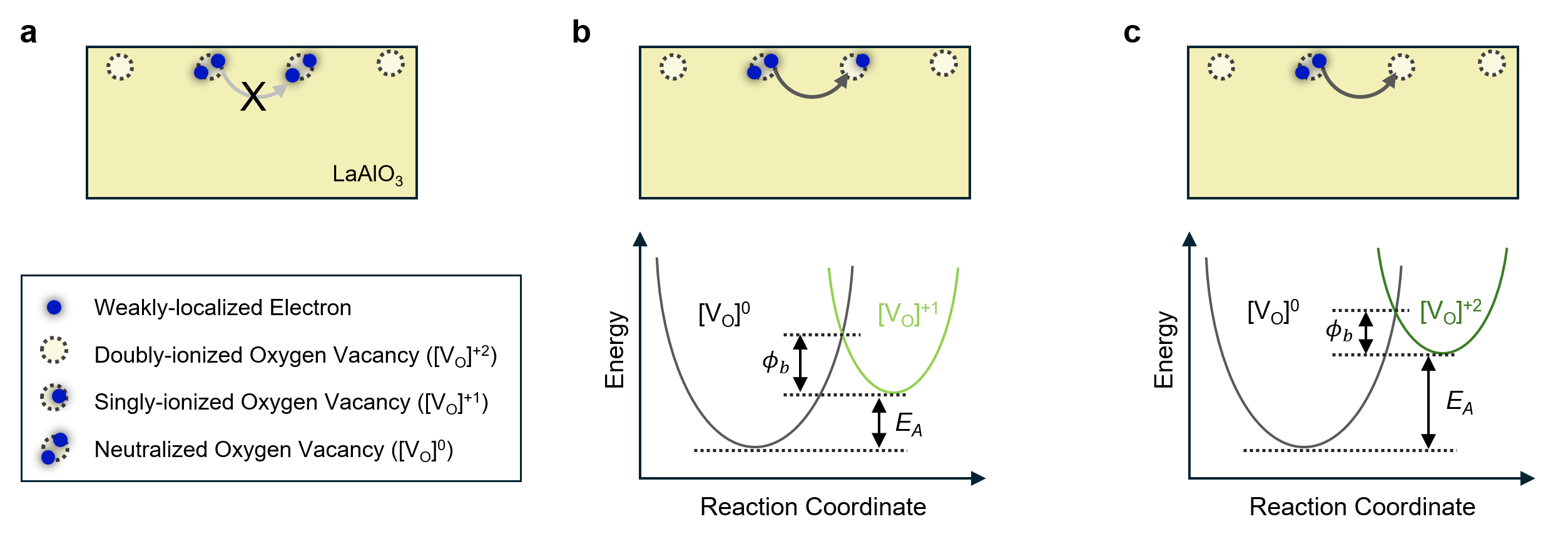


**Figure S2.** Electron hopping between oxygen vacancy defects in LAO. Oxygen vacancies in LAO can exist in three different ionization states: the doubly-ionized ([V_O_]^+2^), the singly-ionized ([V_O_]^+1^), and the neutralized ([V_O_]^0^) states. **(a)** When both neighboring vacancy sites are in the neutralized state, electron hopping does not occur. **(b)** If a neighboring vacancy site can accept an electron, the electron hopping probability becomes non-zero. It should be noted that, since the energy level of the [V_O_]^+1^ state is higher than that of the [V_O_]^0^, additional energy, the activation energy (*E_A_*), is required for the hopping process. Furthermore, this process involves quantum tunneling through an energy barrier imposed by the surrounding lattice, and the height of this barrier is another critical factor influencing the hopping probability and the resulting charge diffusivity. **(c)** The energy level of the [V_O_]^+2^ is slightly higher than that of the [V_O_]^+1^. Therefore, a direct transition from [V_O_]^0^ to [V_O_]^+2^ , which corresponds to the simultaneous hopping of two electrons, requires a higher energy.


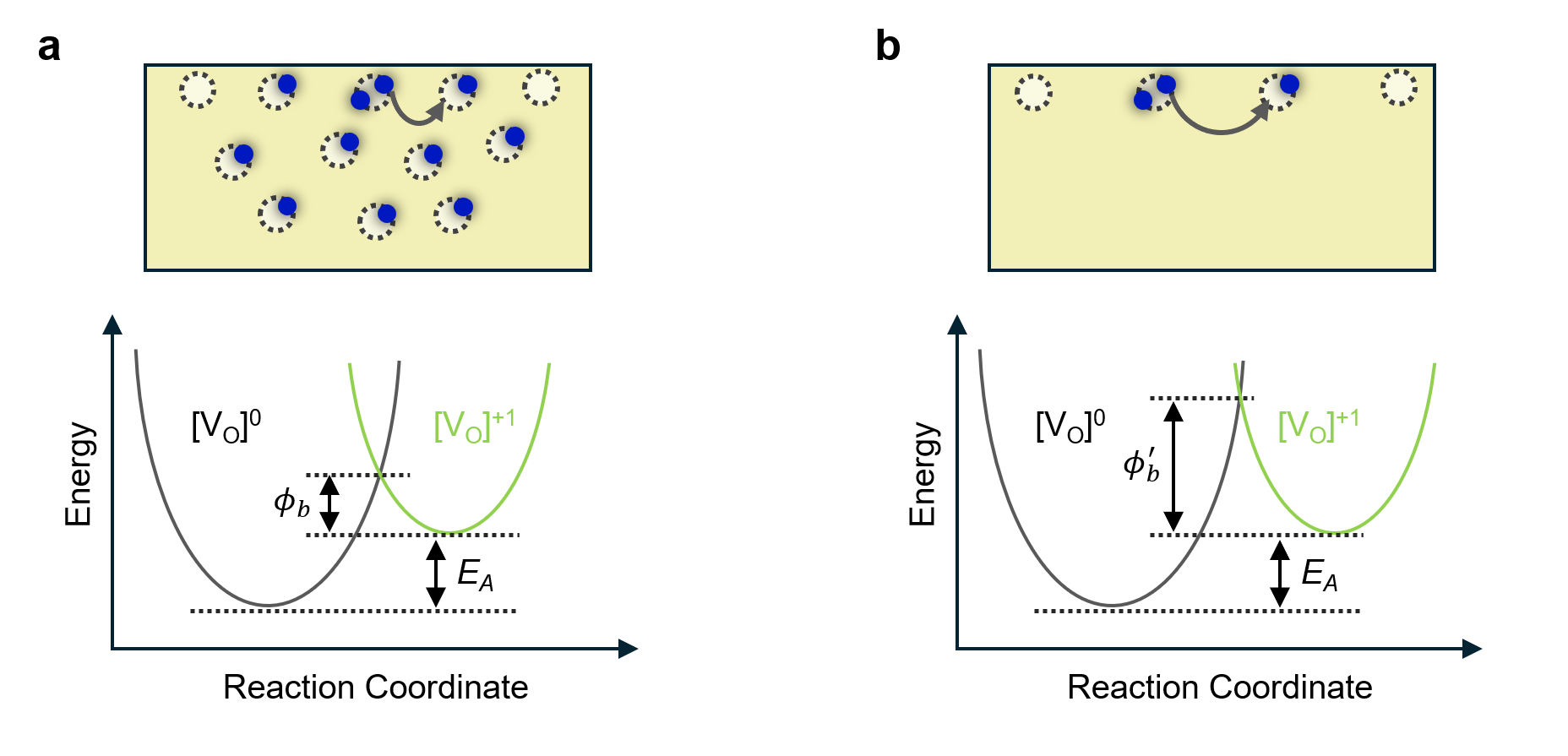


**Figure S3.** Tunneling barrier height ($\phi_{b}$) and activation energy (*E_A_*) for electron hopping depending on oxygen deficiency in LAO thin film. **(a)** In LAO thin films with a high density of oxygen vacancies, the spacing between vacancies becomes shorter and more uniform. In this case, although the activation energy (*E_A_*) remains the same, the barrier height can be reduced, resulting in an increased hopping probability. This, in turn, enhances electron diffusivity at room temperature. **(b)** In contrast, when the oxygen deficiency in LAO is low, the vacancies tend to accumulate near the surface. Under these conditions, both the tunneling distance and the barrier height ($\phi_{b}^{'}$) increase, leading to a significant reduction in the hopping probability.


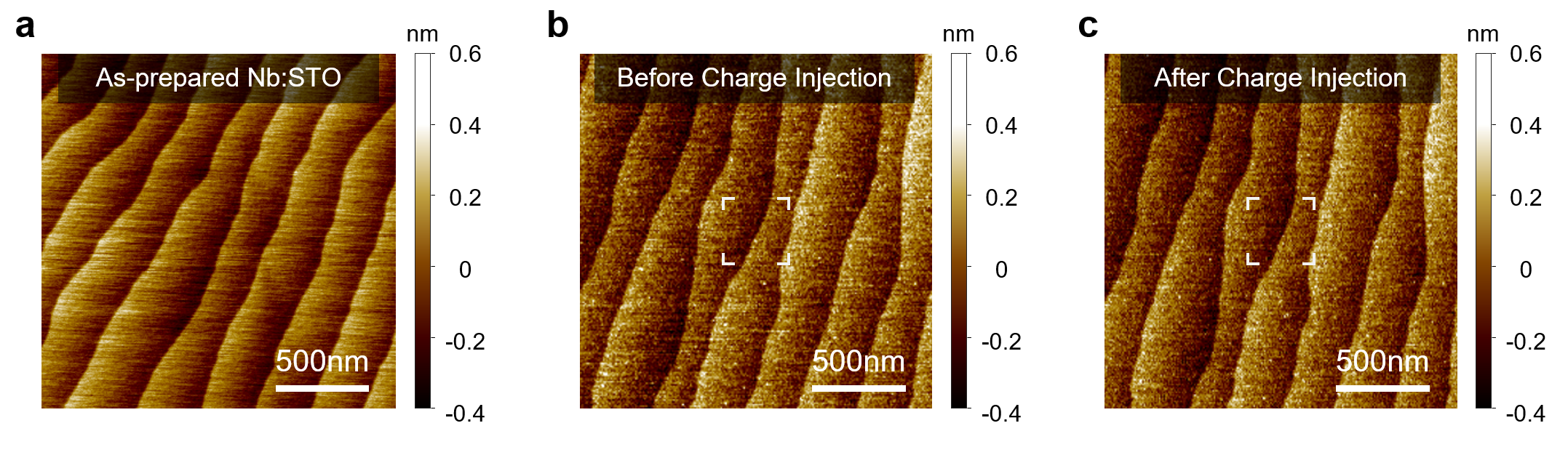


**Figure S4.** Surface topography before and after charge injection by *V_Inj_* of +2.5 V. **(a)** Atomic force microscopy (AFM) image measured on the as-treated Nb:STO (001) substrate. **(b)** AFM image of the as-grown LAO/Nb:STO heterostructure. The atomically smooth step-and-terrace structure of the substrate is well preserved on the LAO thin film, indicating its high quality. **(c)** AFM image measured at the same location after the charge injection process with *V_Inj_* of +2.5 V, the highest *V_Inj_* used in this study. The surface topography remains unchanged after charge injection, confirming that the observed changes in surface potential are not associated with surface adsorbates or extrinsic bulk defects.


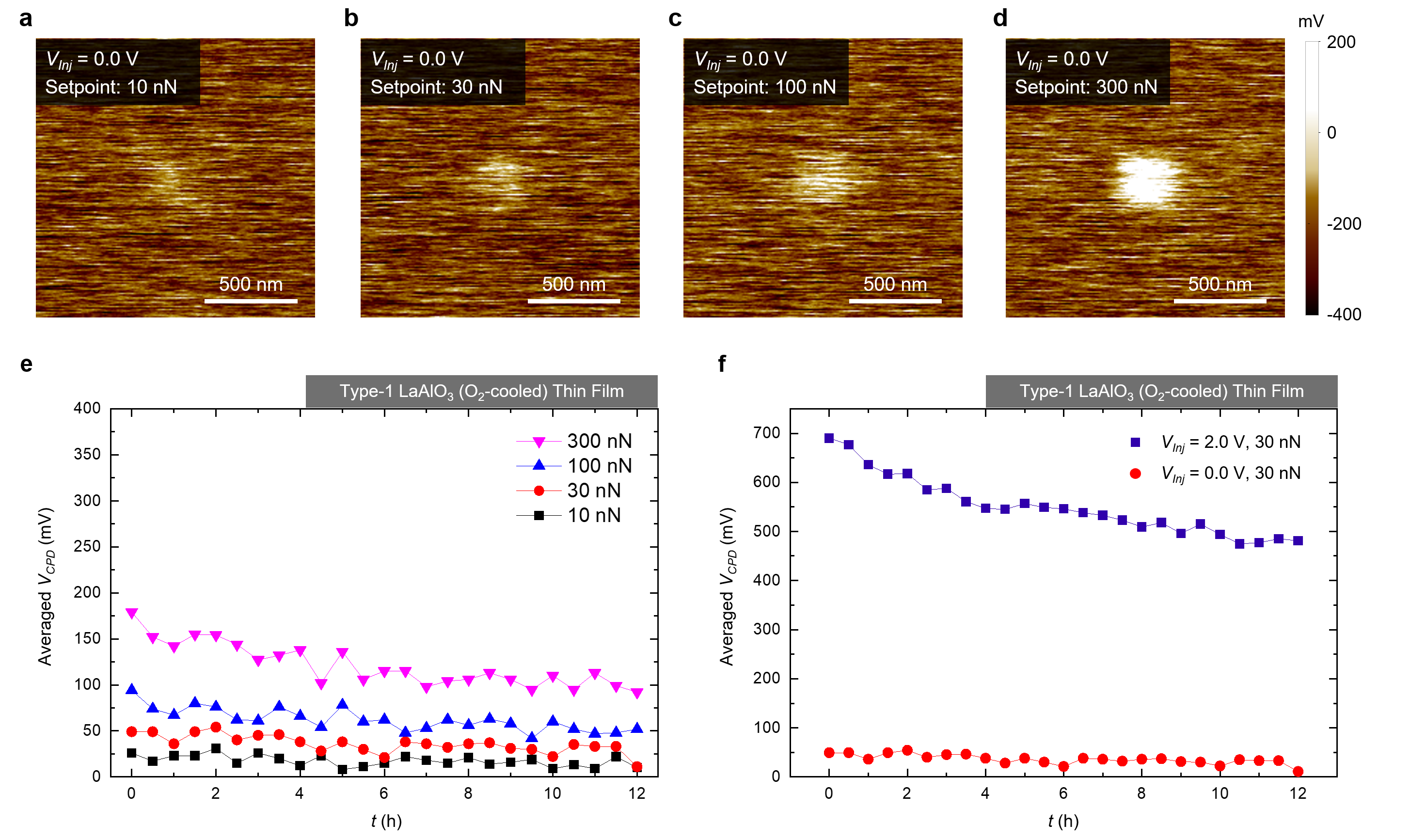


**Figure S5.** Surface potential change induced by mechanical scans. **(a-d)** KPFM images measured after charge injection processes with no bias voltage (i.e., *V_Inj_* = 0 V) and different setpoints (i.e., the contact forces). It is clearly seen that, even with no bias voltage, the mechanical scan with the AFM tip induces a small change in the surface potential on LAO. This increase in *V_CPD_* is attributed to the oxygen vacancy migration by flexoelectric field.^[1-2]^ The mechanical contact between the AFM probe tip and the LAO surface leads to the local structural distortion, inducing the flexoelectric field around the contact area. The flexoelectric field induced during the mechanical scan is oriented in a way that drives oxygen vacancies away from the scanned region, consistent with the observed increase in *V_CPD_*. **(e)** Time-dependent evolution of the averaged *V_CPD_* within the charge-injected regions. Although the magnitude of the mechanically injected charges is relatively small, they were stably retained over 12 hours. **(f)** Time-dependent evolution of *V_CPD_* under the same contact force of 30 nN, but with different *V_Inj_*. From the initial measurements, the *V_CPD_* signal generated solely by mechanical loading corresponds to only ~7.1% of that obtained under an injection bias of *V_Inj_* = +2.0 V. These results show that, while the flexoelectric effect does influence charge retention, it does not represent a major contribution under our experimental conditions.


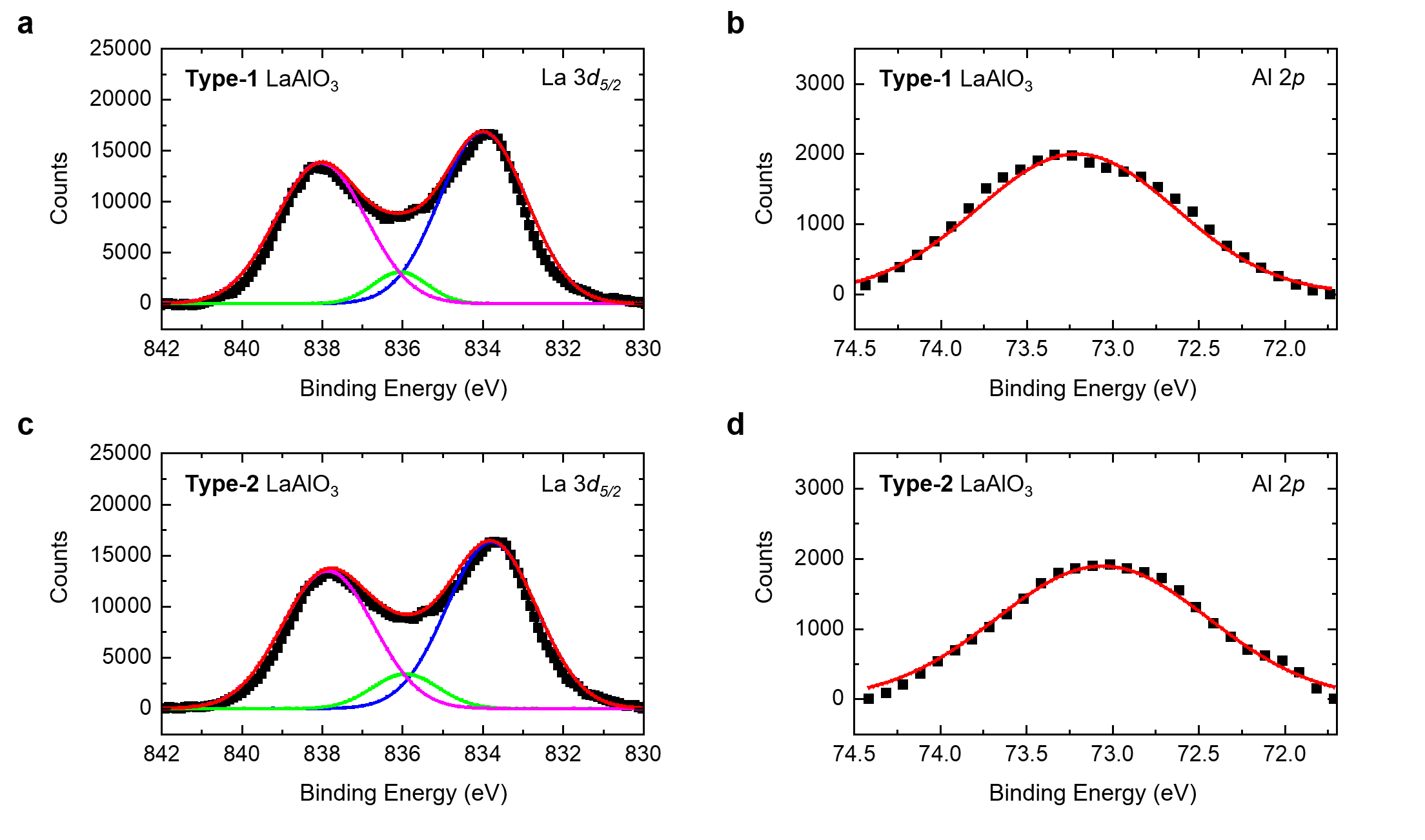


**Figure S6.** Cation stoichiometry analyses by X-ray photoelectron spectroscopy (XPS). **(a)** XPS La 3*d_5/2_* core-level spectrum for the Type-1 LAO thin film, showing two main peaks centered at 834.0 eV (blue) and 838.0 eV (magenta). A minor satellite peak at 836.1 eV (green) is attributed to La atoms associated with oxygen vacancies. **(b)** XPS Al 2*p* core-level spectrum (centered at 73.21 eV) for the Type-1 LAO sample. The red curve represents the fitted result. **(c, d)** XPS core-level spectra of (c) La 3*d_5/2_* and (d) Al 2*p* for the Type-2 LAO thin film. Cation stoichiometry for each sample was calculated using the XPS peak areas and the relative sensitivity factors for La and Al.^[3]^ The La:Al ratios were estimated to be 0.991:1 and 0.997:1 for the Type-1 and Type-2 samples, respectively. These results indicate that while oxygen deficiency differs clearly between the two types of samples, it is not severe enough to cause cation off-stoichiometry or crystalline degradation.^[4]^


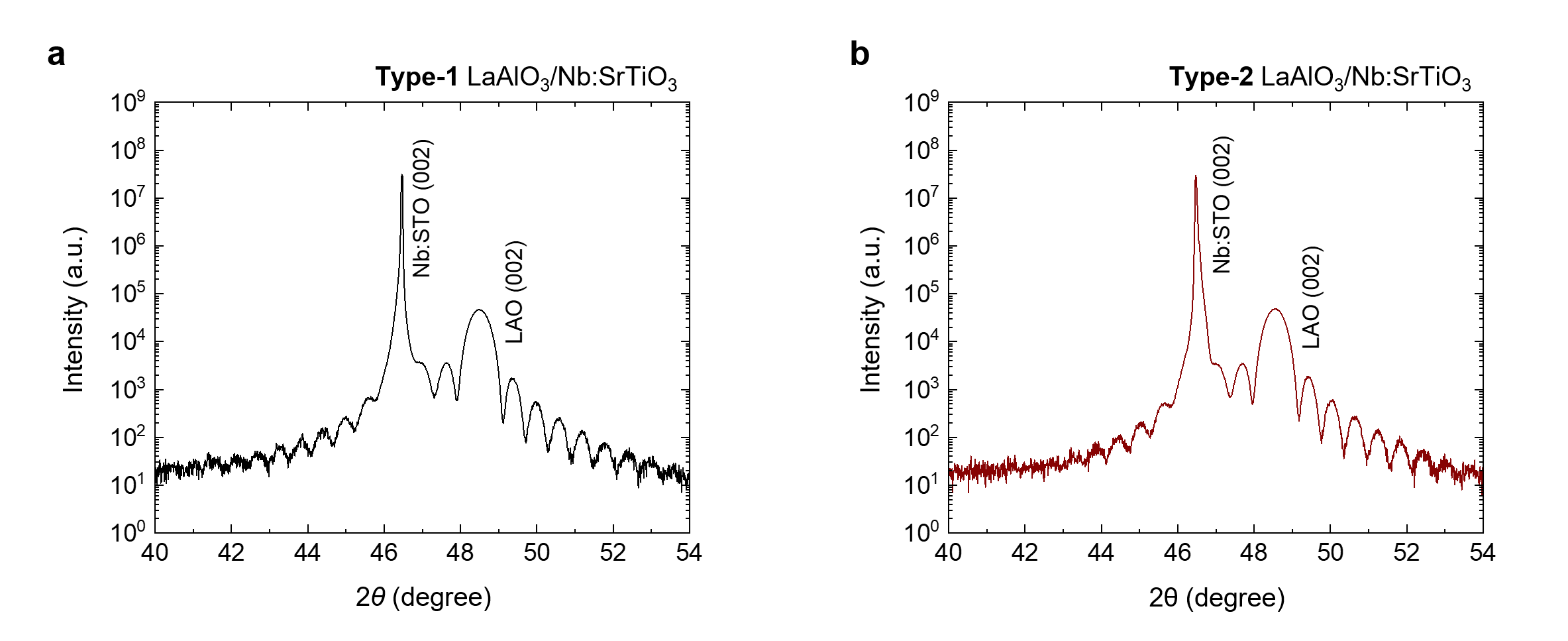


**Figure S7.** Structural analyses of LAO/Nb:STO heterostructures by X-ray diffraction (XRD). **(a, b)** XRD *θ*-2*θ* scans of (a) Type-1 and (b) Type-2 LAO/Nb:STO heterostructures. Both LAO films show clear LAO (002) diffraction peaks and well-defined thickness fringes, indicating high crystalline quality and coherent epitaxial growth on Nb:STO (001) substrates.


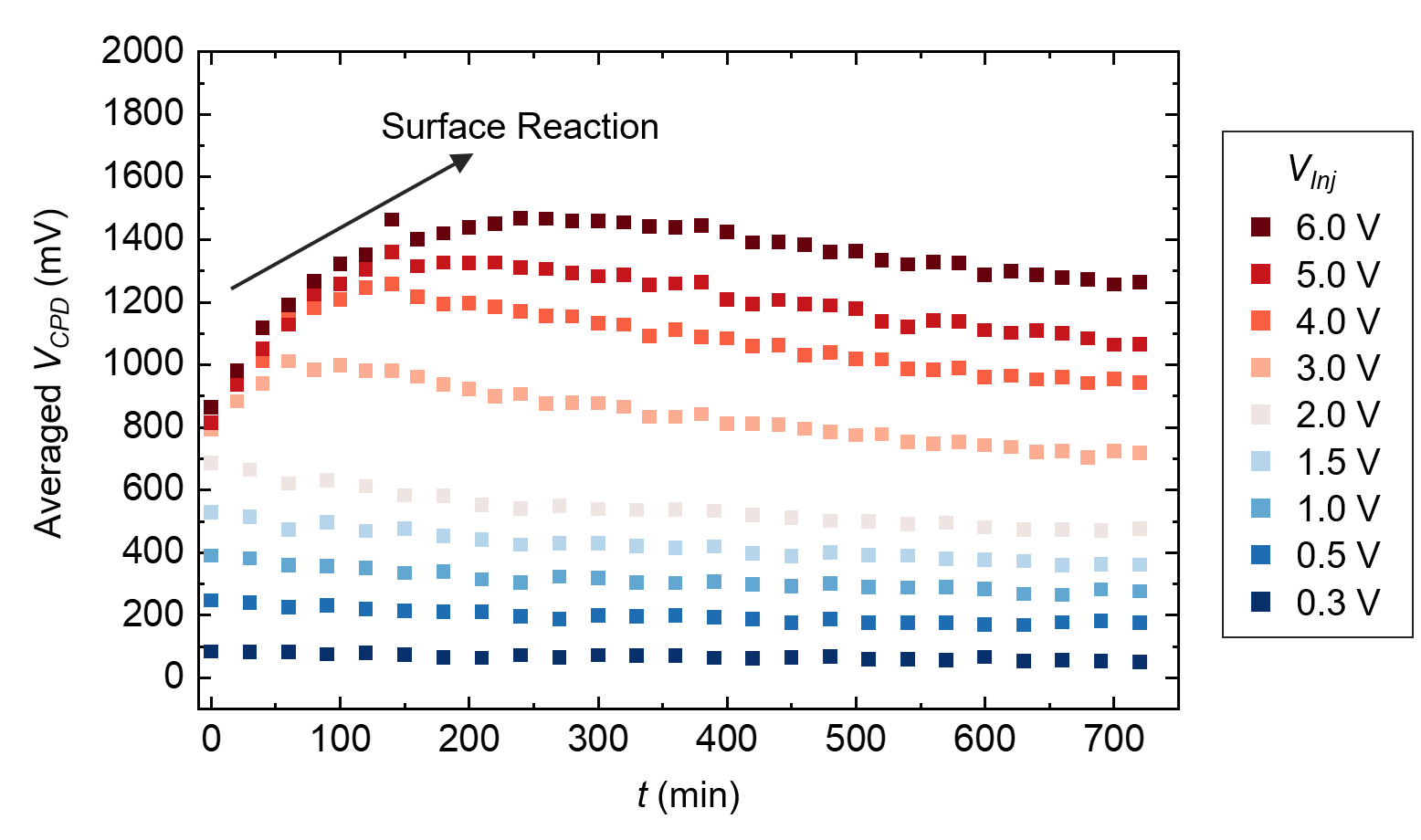


**Figure S8.** Time-dependent evolution of the averaged KPFM signals obtained from the Type-1 sample with different *V_Inj_*. After charge injection with a constant bias voltage (*V_Inj_*), the change in *V_CPD_* induced by the injected charges generally exhibited a monotonic decrease over time. However, when *V_Inj_* exceeded +3.0 V, a temporary increase in *V_CPD_* was observed during the initial 100 to 200 min before beginning to decay. This anomalous behavior is presumably caused by surface reactions initiated by the strong electric field concentrated at the AFM probe tip during the injection process​. Specifically, the adsorption of self-dissociated water molecules and other ambient polar molecules onto the LAO surface can cause the transient rise in *V_CPD_*. To exclude such extrinsic effects, we limited *V_Inj_* to +2.5 V, under which no such abnormal behavior was observed.


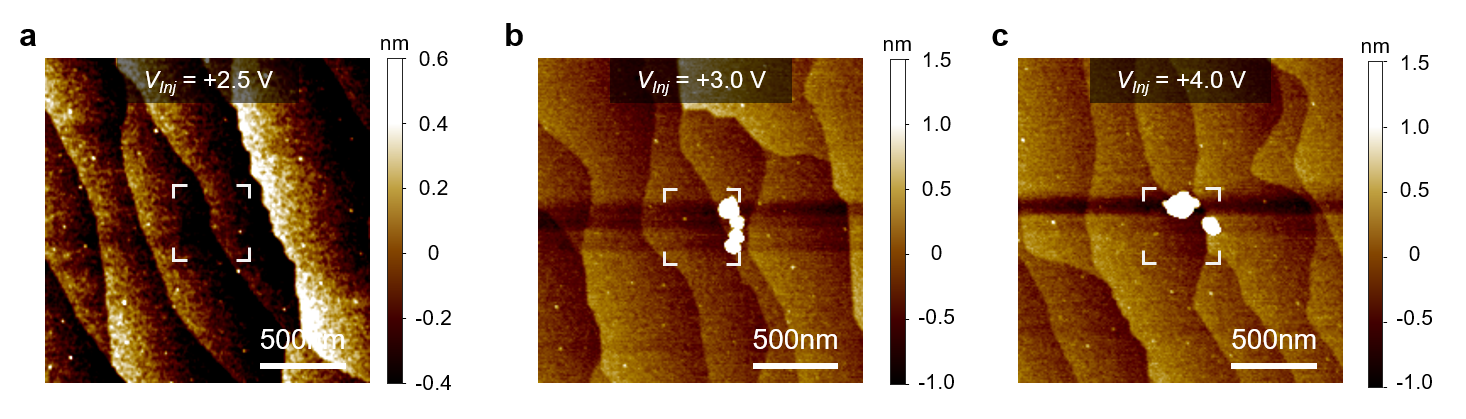


**Figure S9.** **(a-c)** AFM topography image measured on LAO surface after charge injection with *V_Inj_* of (a) +2.5 V, (b) +3.0 V, and (c) +4.0 V. The white markers indicate the locations where the charge injection was performed. We observed that charge injections with *V_Inj_* > +2.5 V often resulted in surface roughening, indicating the adsorption of environmental molecules. To avoid such extrinsic effect, we restricted our analysis to data obtained with *V_Inj_* ≤ +2.5 V. It should be noted, however, that the threshold voltage at which extrinsic effects emerge may vary across different materials, owing to numerous perturbations, including variations in the effective magnitude of local electric field as well as external factors such as surface contamination, airborne dust and humidity, and subtle differences in the AFM tip contact area.


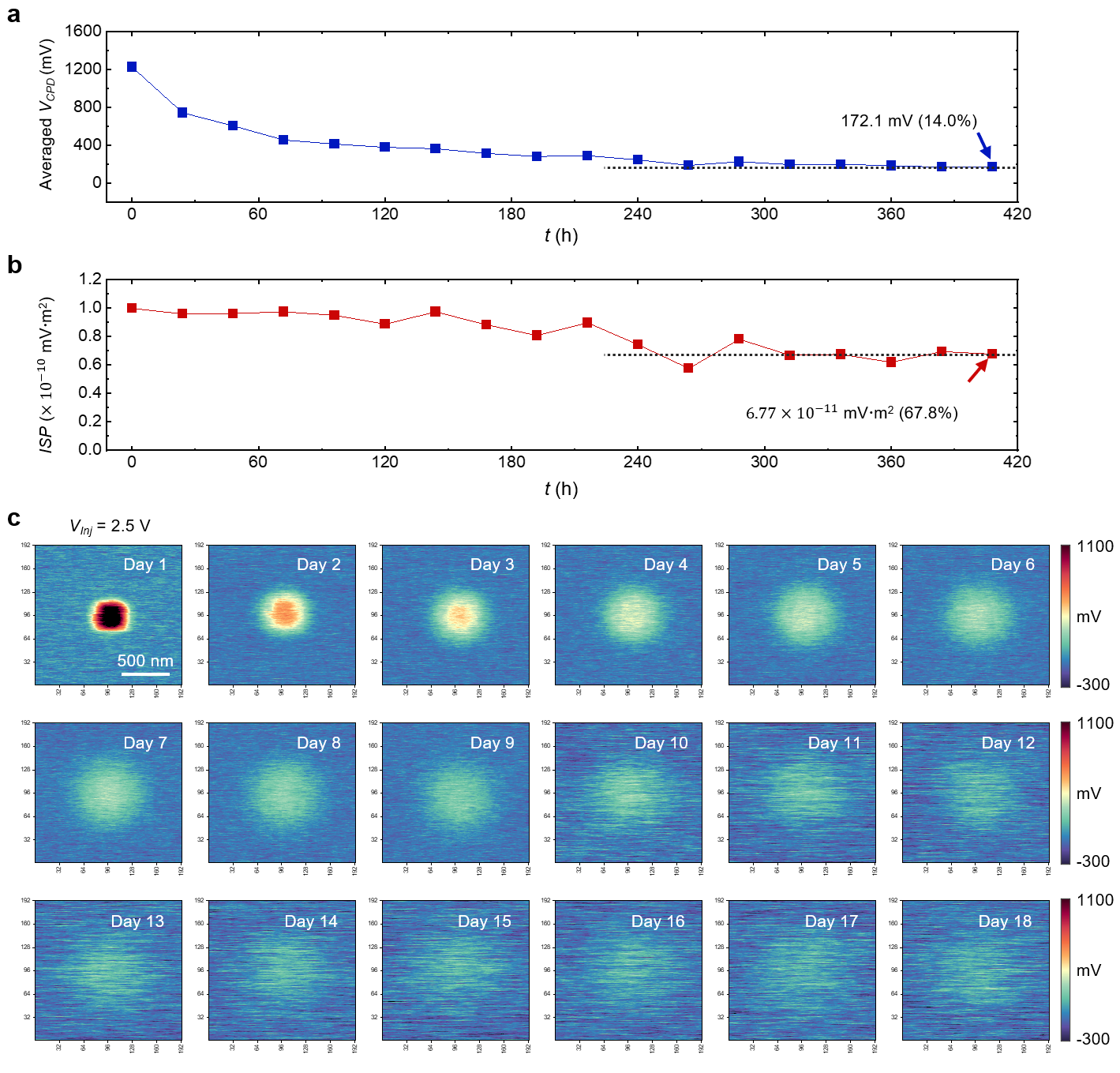


**Figure S10.** Charge retention characteristics in Type-1 LAO/Nb:STO heterostructure over an extended period. **(a)** Long-term monitoring of the injected charges in the Type-1 sample using KPFM. Charge injection was performed on the LAO surface by *V_Inj_* of +2.5 V. These measurements were conducted independently from the experiment shown in Figure 4, starting from the beginning. Thus, this result demonstrates that the long-term charge retention behavior in LAO is highly reproducible. **(b)** Time-dependent evolution of *V_CPD_* values averaged within the central 25 × 25 pixels of the charge-injected region. Approximately 19.9% of the initial *V_CPD_* value was retained 10 days after the initial charge injection, and at least 14.0% remained even after 17 days. **(c)** Time-dependent integrated surface potential (ISP). The *ISP* value showed a gradual decrease after the initial charge injection. Nevertheless, over 67.8% of the injected charges remained on the surface even after 17 days, clearly demonstrating the exceptional charge retention capability of the Type-1 LAO sample.


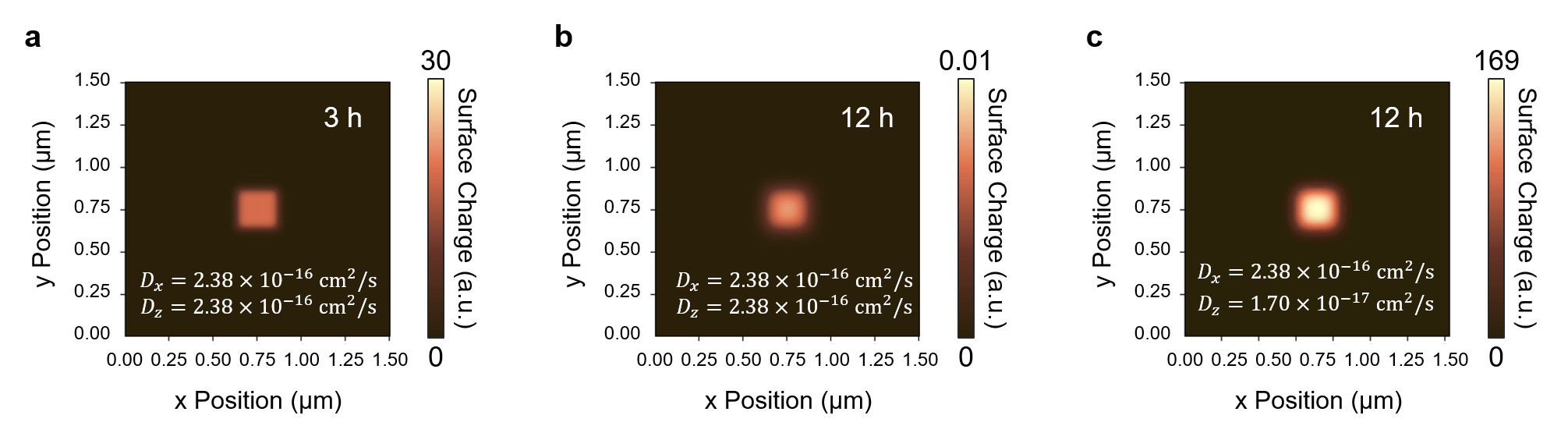


**Figure S11.** Simulated images of remnant surface charges at several hours after charge injection. **(a)** Rescaled image of the data at 3 h from Figure 5b, **(b)** Rescaled image of the data at 12 h from Figure 5b.


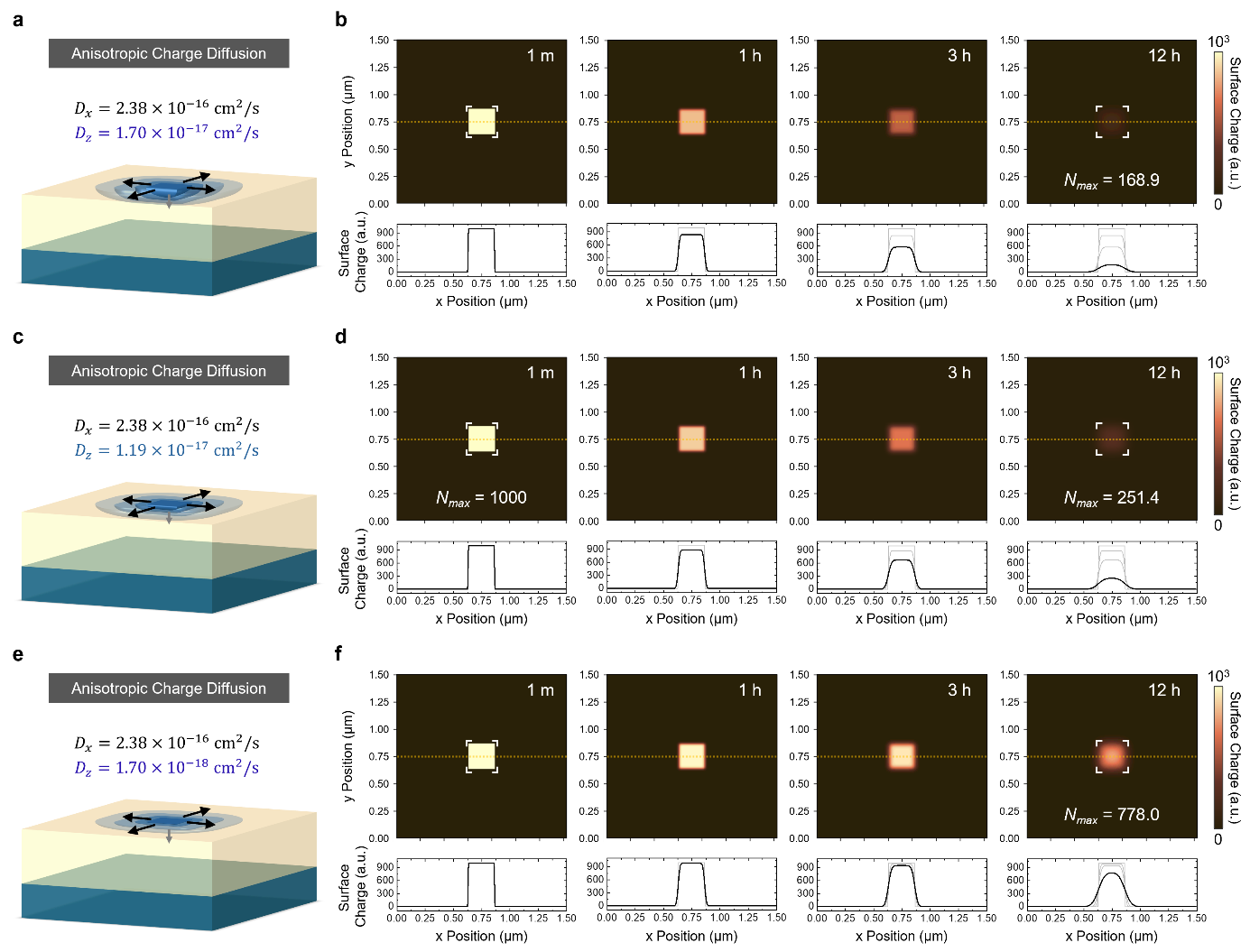


**Figure S12.** Charge retention under different diffusion anisotropies. **(a)** Schematic illustration of anisotropic charge diffusion. The in-plane and out-of-plane diffusivities are set to be $D_{x}=2.38\times{10}^{-16}$cm^2^/sec and $D_{z}=1.70\times{10}^{-17}$cm^2^/sec, respectively. **(b)** Simulated surface charge distributions at 1 min, 1 h, 3 h, and 12 h after the charge injection. Below each image, line profiles along the yellow dashed line (*y* = 0.75 μm) show the surface charge density as a function of *x*-position. **(c)** Simulation with a slightly reduced out-of-plane diffusivity, $D_{z}=1.19\times{10}^{-17}$ cm^2^/sec. **(d)** Corresponding evolution of charge distribution. **(e)** Simulation with a significantly reduced out-of-plane diffusivity, $D_{z}=1.70\times{10}^{-18}$ cm^2^/sec. **(f)** Corresponding charge distribution evolution, clearly demonstrating improved charge retention. After 12 h, 77.8% of injected charges survived on the film surface. This result confirms that a higher degree of diffusion anisotropy leads to greator retention of surface charges.

**Supplementary Note S1**

**Electronic and ionic contributions to charge retention**

To minimize ionic effects, we limited the *V_Inj_* to < +3.0 V, corresponding to an electric field of ~1.5 MV/cm (parallel‑plate approximation). To quantitatively estimate field-driven ionic drift, we applied the Nernst-Planck framework^[5]^: $\boldsymbol{J}(\boldsymbol{r},t)=-D\nabla n+\mu n\boldsymbol{E}$, with the ionic mobility expressed as $\mu=Dq/kT$. The *J*, *D*, *n*, and *E* represent flux, diffusivity, local oxygen vacancy concentration, and the applied electric field, respectively. Using a previously reported oxygen vacancy diffusivity for perovskite oxides such as SrTiO_3_ ($D\approx{10}^{-17} \mathrm{cm}^{2}/s$)^[6]^, the calculated drift length under our experimental conditions (*V_Inj_* = +2.5 V, *E* ~1.25 MV/cm, Injection time ~30 s, *q* = +2*e*) is only ~0.29 nm. Since LAO exhibits an even higher activation enthalpy for oxygen transport (~0.9 eV), the actual ionic drift at room temperature should be smaller. Therefore, we can conclude that charge injection processes at *V_Inj_* ≤ +2.5 V result in only limited ionic motion in LAO. Although a full quantitative separation of ionic and electronic contributions is challenging, these estimates indicate that the observed KPFM signals in our main datasets are predominantly governed by electronic charge modulation rather than oxygen-vacancy migration.

**Supplementary Note S2**

**Integrated surface potential (ISP) calculation**

Since the measured *V_CPD_* value is directly proportional to the surface charge density, we define the ISP as the sum of *V_CPD_* values multiplied by the pixel area across the entire KPFM image. Specifically, we first subtracted the average background signal, measured on the as-grown LAO surface, from the *V_CPD_* dataset (*i.e.,* 192×192 array). We refer this background-subtracted *V_CPD_* to *ΔV_CPD_.* Next, we excluded all data points with *ΔV_CPD_* values less than 20% of the maximum, in order to isolate the contribution from injected charges and minimize the influence of background fluctuations. We then multiplied each *ΔV_CPD_* value by the unit pixel area of 6.10 × 10^-17^ m^2^ and finally summed the results over all pixels to obtain the ISP.

**Supplementary Note S3**

**Finite-difference simulations of charge diffusion in LAO thin films**

**
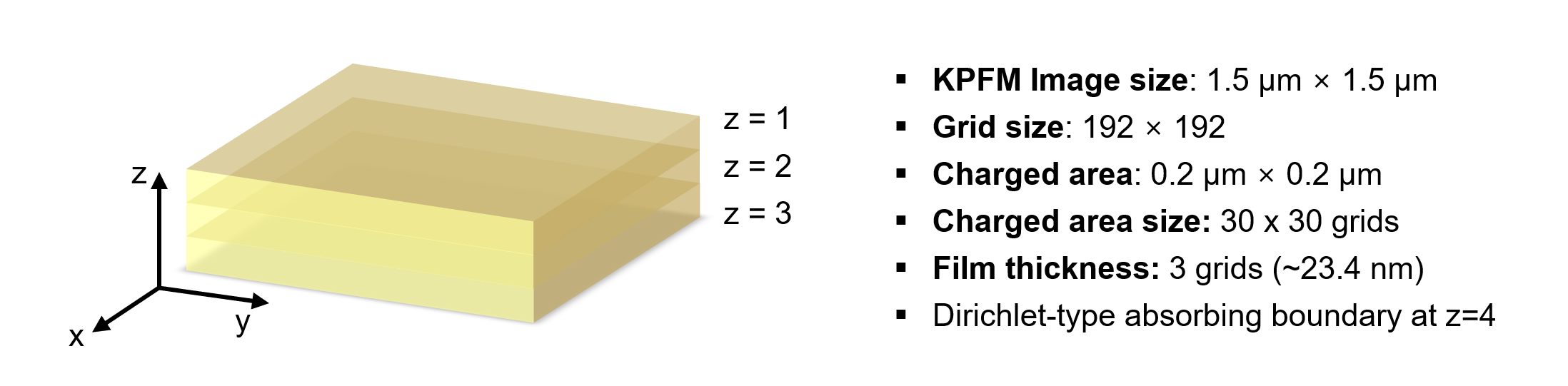
**

**Figure S13.** Material structure and key parameters for simulation of charge diffusion.

This finite-difference simulation^[7]^ was performed by numerically solving the three-dimensional (3D) diffusion equation:

$\frac{\partial C}{\partial t}=D\left( \frac{\partial^{2}C}{{\partial x}^{2}}+\frac{\partial^{2}C}{{\partial y}^{2}}+\frac{\partial^{2}C}{{\partial z}^{2}} \right)$ (Eq. S1)

where *C* represents the density of arbitrary-unit charge on LAO film. The continuous function *C*(*x, y, z, t*) was discretized as $c_{i,j,k}^{n}$, where $x=i\Delta x$, $y=j\Delta y$, $z=k\Delta z$, and $t=n\Delta t$. *n* is a positive integer. The indices *i*, *j*, and *k* range from 1 to 192, corresponding to the spatial resolution used in our KPFM experiments. The $\Delta x$ and $\Delta y$ were set to be 7.81 nm. This spatial resolution is equivalent to that obtained when measuring a 4 μm × 4 μm region at a resolution of 512×512 pixels with AFM. For consistency, $\Delta z$ was also set to be 7.81 nm. Although the actual film thickness is 20 nm (50 unit cells.), the *z*-direction was approximated with 3 discrete layers (i.e., ~23.4 nm). The finite difference approximation yields the following expression:

$\frac{c_{i,j,k}^{n+1}-c_{i,j,k}^{n}\text{ }}{\Delta t}=D_{x}\left\{ \left( \frac{c_{i+1,j,k}^{n}-2c_{i,j,k}^{n}+c_{i-1,j,k}^{n}}{{(\Delta x)}^{2}} \right)\text{ }+\left( \frac{c_{i,j+1,k}^{n}-2c_{i,j,k}^{n}+c_{i,j-1,k}^{n}}{{(\Delta y)}^{2}} \right)\text{ } \right\}+D_{z}\left( \frac{c_{i,j,k+1}^{n}-2c_{i,j,k}^{n}+c_{i,j,k-1}^{n}}{{(\Delta z)}^{2}} \right)$ (Eq. S2)

where $c_{i,j,k}^{n+1}$ is the charge density at the next time step. To capture the spatial anisotropy of charge diffusion in 3D, we employed distinct diffusivities for the in-plane (*D_x_*) and out-of-plane (*D_z_*) directions. The *D_x_* value was set to be $D_{x}\sim2.38\times{10}^{-16}$ cm^2^/sec, as estimated from the experimental results. As a starting point, *D_z_* was set equal to *D_x_* ($2.38\times{10}^{-16}$ cm^2^/sec) and then gradually reduced. The exact coefficients are not critically important here, as they can vary with sample-to-sample differences and practical perturbations. Instead, *D_z_* was iteratively adjusted until the simulated charge retention performance closely reproduced the experimental observations. The time step *Δt* was determined based on the stability condition of the explicit finite difference method for 3D anisotropic diffusion. According to the von Neumann stability criterion, the sum of the diffusion terms in all directions must remain below a critical threshold in each update step. Accordingly, the time step was set as:

$\Delta t=\frac{1}{2D_{x}\left( \frac{1}{{\Delta x}^{2}}+\frac{1}{{\Delta y}^{2}} \right)+{2D}_{z}\left( \frac{1}{{\Delta z}^{2}} \right)}$ (Eq. S3)

At the initial state, arbitrary-unit charges were assumed to be injected at the center of the surface layer (i.e., *z* = 1). In this way, as shown in Figure 5, the time-dependent charge distribution on the LAO surface (*z* = 1) was evaluated at 60 s (1 m), 3600 s (1 h), 10,800 s (3 h), and 43,200 s (12 h).

**Supplementary Table S1.** Summary of variables and parameters

| Parameter Name | Meaning |
| --- | --- |
| *C* (*x, t*) | The time-dependent spatial distribution of surface charge |
| *D_x_* | In-plane diffusivity [cm^2^/s] |
| *D_z_* | Out-of-plane diffusivity [cm^2^/s] |
| $\tau_{z}$ | The out-of-plane decay time constant for *C* [sec] |
| *P_surface_* (*t*) | The time-dependent surface polarization [mV] |
| $\tau_{1}$ | The fast decay time constant for *P_surface_* [h] |
| $\tau_{2}$ | The slow decay time constant for *P_surface_* [h] |
| $\Gamma$ | The hopping rate of electrons between neighboring oxygen vacancies |
| $\alpha$ | The inverse localization length [m] |
| *d* | The distance between neighboring oxygen vacancies [m] |

**Supplementary Table S2.** Summary of simulation parameters and results

| Charge diffusion isotropy | *D_x_* [cm^2^/s] | *D_z_* [cm^2^/s] | (Simulation)  Relative residual charge [%] after 12 h | (Experimental)  Relative residual charge [%] after 12 h |
| --- | --- | --- | --- | --- |
| Isotropic | $2.38\times{10}^{-16}$ | $2.38\times{10}^{-16}$ | ~0.00007 | 69.71 |
| Anisotropic | $2.38\times{10}^{-16}$ | $1.70\times{10}^{-17}$ | 16.89 | 69.71 |
| Anisotropic | $2.38\times{10}^{-16}$ | $1.19\times{10}^{-17}$ | 25.14 | 69.71 |
| Anisotropic | $2.38\times{10}^{-16}$ | $2.56\times{10}^{-18}$ | 69.58 | 69.71 |
| Anisotropic | $2.38\times{10}^{-16}$ | $1.70\times{10}^{-18}$ | 77.80 | 69.71 |

**Supplementary Table S3.** Charge retention properties of various oxide systems

| Materials | Charge retention property ^a)^ | Reference |
| --- | --- | --- |
| Oxygen-vacancy-engineered LaAlO_3_ | 69.71% of the injected charges were retained after 288 min (12 h).  67.8% of ISP was retained after 420 h. | This work |
| SrTiO_3_/LSAT | ~50% of the injected charges were retained after 180min. | [8] |
| SrTiO_3_/GdScO_3_ | ~25% of the injected charges were retained after 180min. | [8] |
| SiO_2_ | ~45% of the injected charges were retained after 100 min. | [9] |
| Cu-doped ZnO | ~50% of the injected charges were retained after 60 min. | [10] |
| (K,Na)NbO_3_ | Remnant polarization was removed after 250 h. | [11] |
| In-Ga-Zn-O (IGZO) | ~80% of the injected charges were retained after 200 s. | [12] |
| Al_2_O_3_/HfO_2_/Al_2_O_3_ | ~70% of the injected charges were retained after 500 min | [13] |
| CeO_2_/Si/CeO_2_/Si | ~50% of the injected charges were retained after ~400 min. | [14] |

^a)^ As retention properties are reported in diverse ways across the literature, we approximated them into comparable metrics for consistency.

**References**

1. S. Das, B. Wang, Y. Cao, M. Rae Cho, Y. Jae Shin, S. Mo Yang, L. Wang, M. Kim, S. V. Kalinin, L.-Q. Chen, *Nature communications* **2017**, *8* (1), 615.
2. S. M. Park, B. Wang, L.-Q. Chen, T. W. Noh, S. M. Yang, D. Lee, *Applied Physics Reviews* **2021**, *8* (4).
3. M. Lee, Y. Kim, S. H. Mo, S. Kim, K. Eom, H. Lee, *Small* **2024**, *20* (25), 2309851.
4. M. Warusawithana, C. Richter, J. A. Mundy, P. Roy, J. Ludwig, S. Paetel, T. Heeg, A. Pawlicki, L. F. Kourkoutis, M. Zheng, *Nature communications* **2013**, *4* (1), 2351.
5. R. Meyer, R. Liedtke, R. Waser, *Applied Physics Letters* **2005**, *86* (11).
6. A. Paladino, *Journal of the American Ceramic Society* **1965**, *48* (9), 476.
7. K. E. Novik, P. V. Coveney, *The Journal of Chemical Physics* **1998**, *109* (18), 7667.
8. L. Iglesias, A. Gomez, M. Gich, F. Rivadulla, *ACS Applied Materials & Interfaces* **2018**, *10*, 35367.
9. C. Dumas, L. Ressier, J. Grisolia, A. Arbouet, V. Paillard, G. BenAssayag, S. Schamm, P. Normand, *Microelectronic Engineering* **2008**, *85* (12) 2358.
10. M. F. Wong, T. S. Herng, Z. Zhang, K. Zeng, J. Ding, *Applied Physics Letters* **2010**, 97 (23), 232103.
11. M. Sheeraz, C. W. Ahn, N. X. Duong, S. Hwang, J. Jang, E. Kim, Y. K. Kim, J. Lee, J. S. Jin, J. Bae, M. H. Lee, H. Han, G. Kim, S. Cho, T. K. Song, S. M. Yang, S. D. Bu, S. Baek, S. Choi, I. W. Kim, T. H. Kim, *Advanced Science* **2024**, *11* (48), 2408784.
12. S. Lee, Sungsik, *Scientific Reports* **2024**, *14* (1), 11863.
13. Y. Lo, K. Liu, C. Cheng, J. Wu, C. Hou, T. Wu, *Applied Physics Letters* **2009**, *94*, 082901.
14. J. T. Jones, P. M. Bridger, O. J. Marsh, T. C. McGill, *Applied physics letters* **1999**, *75* (9), 1326.
